# Application of omics in the diagnosis, prognosis, and treatment of acute myeloid leukemia

**DOI:** 10.1186/s40364-024-00600-1

**Published:** 2024-06-10

**Authors:** Zhiyu Zhang, Jiayi Huang, Zhibo Zhang, Hongjie Shen, Xiaowen Tang, Depei Wu, Xiebing Bao, Guoqiang Xu, Suning Chen

**Affiliations:** 1 National Clinical Research Center for Hematologic Diseases, Jiangsu Institute of Hematology, the First Affiliated Hospital of Soochow University, Suzhou, China; 2 Jiangsu Key Laboratory of Neuropsychiatric Diseases and College of Pharmaceutical Sciences, Jiangsu Province Engineering Research Center of Precision Diagnostics and Therapeutics Development, Jiangsu Key Laboratory of Preventive and Translational Medicine for Geriatric Diseases, Suzhou Key Laboratory of Drug Research for Prevention and Treatment of Hyperlipidemic Diseases, Soochow University, Suzhou, 215123 Jiangsu China; 3 Suzhou International Joint Laboratory for Diagnosis and Treatment of Brain Diseases, College of Pharmaceutical Sciences, Soochow University, Suzhou, 215123 Jiangsu China; 4 MOE Key Laboratory of Geriatric Diseases and Immunology, Suzhou Medical College of Soochow University, Suzhou, 215123 Jiangsu Province China

**Keywords:** AML, Omics, Biomarker, Risk stratification, Targeted therapy, Venetoclax, FLT3, Menin inhibitor

## Abstract

Acute myeloid leukemia (AML) is the most frequent leukemia in adults with a high mortality rate. Current diagnostic criteria and selections of therapeutic strategies are generally based on gene mutations and cytogenetic abnormalities. Chemotherapy, targeted therapies, and hematopoietic stem cell transplantation (HSCT) are the major therapeutic strategies for AML. Two dilemmas in the clinical management of AML are related to its poor prognosis. One is the inaccurate risk stratification at diagnosis, leading to incorrect treatment selections. The other is the frequent resistance to chemotherapy and/or targeted therapies. Genomic features have been the focus of AML studies. However, the DNA-level aberrations do not always predict the expression levels of genes and proteins and the latter is more closely linked to disease phenotypes. With the development of high-throughput sequencing and mass spectrometry technologies, studying downstream effectors including RNA, proteins, and metabolites becomes possible. Transcriptomics can reveal gene expression and regulatory networks, proteomics can discover protein expression and signaling pathways intimately associated with the disease, and metabolomics can reflect precise changes in metabolites during disease progression. Moreover, omics profiling at the single-cell level enables studying cellular components and hierarchies of the AML microenvironment. The abundance of data from different omics layers enables the better risk stratification of AML by identifying prognosis-related biomarkers, and has the prospective application in identifying drug targets, therefore potentially discovering solutions to the two dilemmas. In this review, we summarize the existing AML studies using omics methods, both separately and combined, covering research fields of disease diagnosis, risk stratification, prognosis prediction, chemotherapy, as well as targeted therapy. Finally, we discuss the directions and challenges in the application of multi-omics in precision medicine of AML. Our review may inspire both omics researchers and clinical physicians to study AML from a different angle.

## Introduction

 Progressions in high-throughput technologies, including genomics, transcriptomics, proteomics, and metabolomics, have started to enable precision medicine at the comprehensive molecular level [1]. Individually, each of the omics technologies has been utilized widely in clinical practice and clinical studies of a variety of diseases, including hematological malignancies. However, each omics alone cannot accurately reflect the entire biological complexity of the disease. Therefore, the integration of multiple omics technologies, i.e., multi-omics, has recently emerged to capture a comprehensive landscape of diseases. The definitions and advantages of different omics are listed in Table 1.

**Table 1:** Definitions and advantages of different types of omics

	Definition	Advantages in AML studies
Genomics	Genetic mapping and DNA sequencing of sets of genes or the complete genomes.	The first and most advanced omics technology. The cost of NGS has decreased significantly and it is now a routine approach in diagnosis and classification of AML.
Transcriptomics	Detection and quantification of all RNA in a sample.	Targeted RNA-seq and bulk RNA-seq have relatively low cost. It has been a requisite for the discovery of diagnostic biomarkers for rare hematological diseases.
Proteomics	Identification and quantification of proteins, post translational modifications, and protein interactions.	Enables direct detection and characterization of the products of genomic aberrations.
Metabolomics	Identification and quantification of small molecular metabolites.	Being the most downstream omics, it is also the closest to the phenotype and can reflect instant changes to drugs.
Multi-omics	Integration of more than one omics technology.	Comprehensively demonstrate the complexity of molecular events in the disease states.

**Fig. 1:**
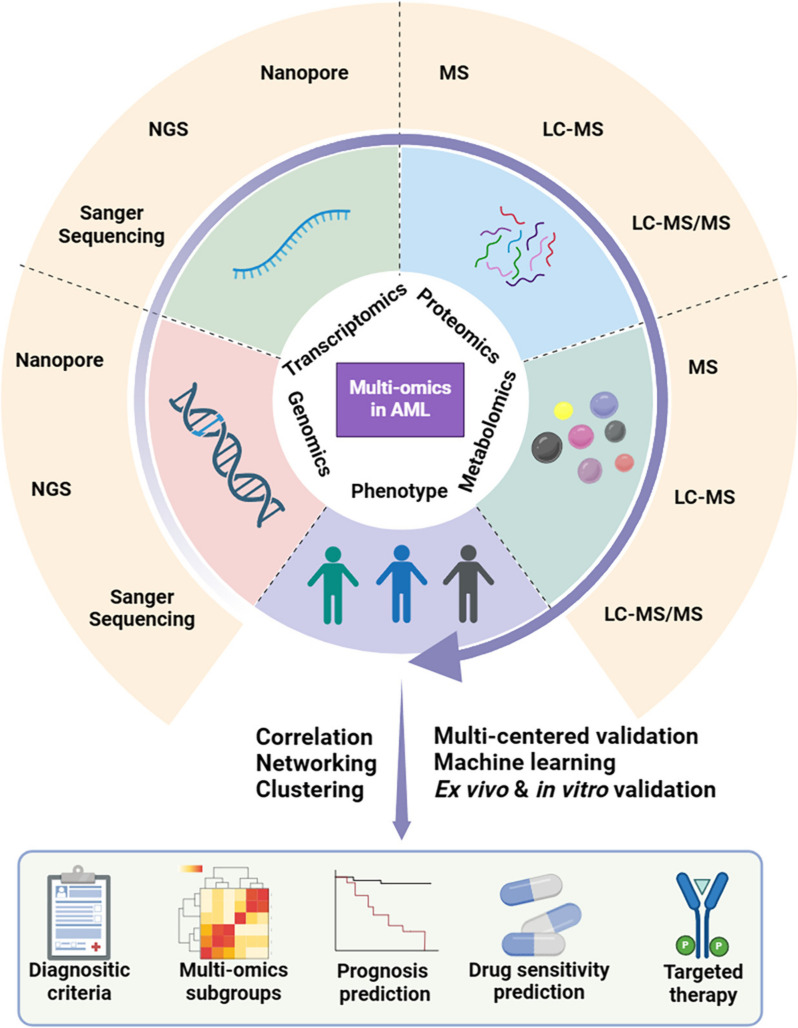
Multi-omics approaches in acute myeloid leukemia (AML) studies. Integrating data from genomics, transcriptomics, proteomics, metabolomics, and clinical phenotypes in different research fields of AML, including diagnosis, molecular subgroups, prognosis, prediction of drug sensitivity, and drug target discovery. Statistical analyses should be performed for the omics data to explore their correlations to clinical manifestations. The identified biomarkers or potential targets need further validation in independent cohorts in vitro or ex vivo

 Acute myeloid leukemia (AML) is a heterogeneous malignant disease characterized by bone marrow (BM) infiltration with leukemic blasts [2]. With the implementation of new treatment strategies over the past years, the 5-year survival rate of AML continued to improve and is now around 28%, but the long-term survival remains dismal [3]. The original diagnostic and classification criterias were based on the degree of maturation of leukemia cells [4]. In the latest edition of the World Health Organization (WHO) classification, AML with defined genetic abnormalities was classified as a specific subtype, partly eliminating the 20% blast cutoff and emphasizing cytogenetic aberrations and mutational profiles [5]. Therefore, prognostic stratifications and selections of therapeutic strategies are mainly determined by mutations and cytogenetic abnormalities [6], and genomics has been extensively studied in AML (Fig. 1). A recent review by Eisfeld et al. [7]. has thoroughly illustrated the genetics, epigenetics, and genomic characteristics of AML and their influence on the treatment and disease prognosis.

Despite comprehensive and instructive genomic information, there is an inconsistency between the actual outcomes and the current risk stratification, especially for the intermediate-risk subgroup [8]. We also noticed that some patients, particularly elderly ones, were classified into the favorable-risk group but yielded poor outcomes under standard treatment [9, 10]. Myelodysplastic syndrome (MDS) is a slowly progressing clonal heterogeneous malignancy with a median survival of 5 years [11]. However, 30–40% of MDS patients eventually progress to secondary AML (sAML) and the survival time is less than 6 months, which is even shorter than *de novo* AML [12]. Although mutations like *TP53* may suggest a higher chance of leukemic transformation, there is still a lack of reliable markers for predicting the transformation from MDS to sAML [13].

**Fig. 2:**
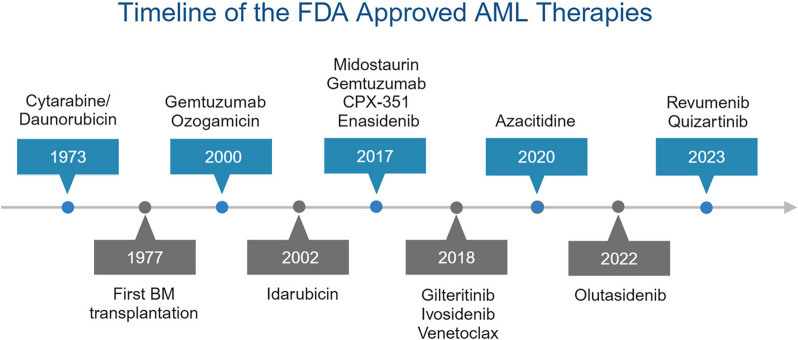
History of the FDA-approved therapies for AML. Since Cytarabine/Daunorubicin (7+3) and hematopoietic stem cell transplantation (HSCT) were approved by the FDA in 1973 and 1977, respectively, no significant progress was made until 2000. Since then, targeted therapies have been developed quickly partly owing to the high-throughput sequencing technologies which helped dissect signaling pathways and identify possible drug targets. FLT3 (Fms related receptor tyrosine kinase 3) inhibitor midostaurin, IDH2 (Isocitrate dehydrogenase 2) inhibitor enasidenib and IDH1 inhibitor ivosidenib have contributed to the significant improvement in patient survival. New generations of these three targeted drugs were also developed, including the newly approved IDH1 inhibitor Olutasidenib and FLT3 inhibitor Quizartinib. The emerging BCL-2 inhibitor venetoclax and menin inhibitor revumenib also have a promising future

 The standard chemotherapy “7 + 3” has been the fundamental treatment for AML ever since its approval in 1973 (Fig. 2). Resistance to standard chemotherapy remains a significant problem, and the main reasons for the resistance include the alternation of genes, mRNA, and proteins, and the aberrant activation of related signaling pathways [14]. The next-generation sequencing (NGS) approaches identified many disease-related mutations and emerging drugs targeting some mutations have greatly improved the survival of AML patients compared with chemotherapy alone. For AML patients with *FLT3* mutation, the FDA (Food and Drug Administration) approved midostaurin in 2017 [15]. Gilteritinib and quizartinib were approved for relapsed/refractory (R/R) AML patients with FLT3 mutation in 2018 and 2019, respectively [16, 17]. In addition to FLT3, other receptor tyrosine kinases (RTKs) like KIT are targets of RTK inhibitors such as midostaurin, sorafenib, dasatinib, and bemcentinib [18, 19]. Besides, the inhibitors of IDH2 (enasidenib) and IDH1 (ivosidenib) were also approved for the treatment of R/R AML with corresponding mutants in 2017 and 2018, respectively [20, 21]. Recently, the approval of venetoclax targeting BCL-2 had significantly improved outcomes of newly diagnosed AML ineligible for intensive chemotherapy [22]. Presenting satisfactory efficacies in AML patients with NPM1 mutation or MLL rearrangement [23, 24], one of the menin inhibitors revumenib was approved by the FDA as an “orphan drug” for treating AML in October 2023. However, there are still various AML patients resistant to these targeted therapies, and many resistance mechanisms remain unclear, demanding further investigation and solutions to overcome resistance [25, 26].

As a hot tool for studying AML, transcriptomics can provide additional information beyond genomics, and it has already become a requisite for the diagnosis markers of another hematological malignancy: myeloid/lymphoid neoplasms with eosinophilia and tyrosine kinase gene fusions (MLN-TK) [5]. RNA-seq could help reclassify and risk-stratify AML based on gene expression and identify many novel fusion genes [27, 28]. Furthermore, the single-cell RNA sequencing (scRNA-seq) technique has made it possible to study the cellular components and cellular hierarchies of the AML microenvironment, deepening the understanding of the molecular basis of AML [29, 30]. A myriad of transcriptomics abnormalities have been identified, but most of them have unidentified clinical significance and only very few of them turned out to be therapeutic targets. Although recent transcriptomics studies provide additional information to gene and associated cell functions, the understanding of biological processes of AML is still limited to the molecular level, lacking a deeper insight into its downstream effects such as protein expression, post-translational modifications (PTMs), and the metabolites produced by leukemic cells or altered by drug administering. Molecular subtypes derived from only one molecular platform limit the ability to identify causative nodes and downstream effects that may be potentially treatable [31, 32].

**Fig. 3:**
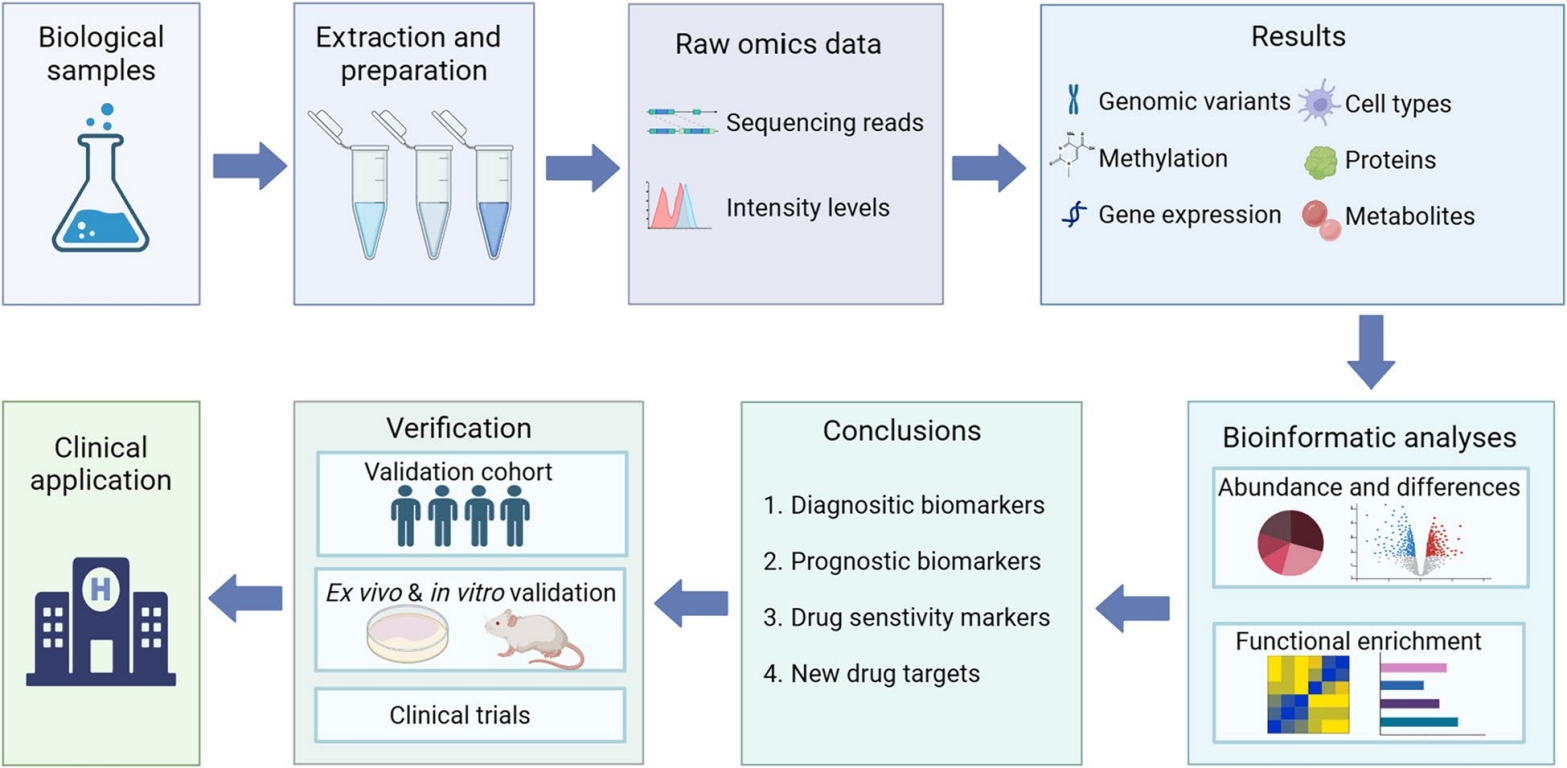
Workflow for omics studies. First, biological samples are collected from patients and healthy controls. DNAs, RNAs, proteins, and metabolites are extracted and prepared for omics analysis. Then, raw omics data are generated through standard protocols, e.g. high-throughput sequencing for transcriptomics and LC-MS for proteomics. After normalization and filtration of raw data and procession through computer software, data for gene expression and protein abundance are obtained. Bioinformatic analyses are then performed to study the expression differences, functions, and their association with possible molecular subtypes. Biomarkers and drug targets are then identified and further verified in patient samples or cell lines

 Therefore, with the growing emphasis on precision medicine and the emergence of new therapeutic options, the molecular subtypes based on genomics, transcriptomics, proteomics, and metabolomics data of AML may be more instructive and better represent the pathological conditions of the disease. The design of both single-omics and multi-omics studies can be complicated (Fig. 3), preparations, analyses, and verifications require a lot of investment and effort. However, a considerable number of studies applying these strategies to AML have been conducted. In this review, we outline the current research on the diagnosis, risk stratifications, biomarker identification, and targeted therapies of AML applying these omics methods.

## Omics in diagnosis of AML

Current diagnostic criteria and classification of AML are mostly based on MICM (morphology, immunology, cytogenetics, and molecular biology). However, as a heterogenous disease, the classification of AML based on morphology, cytogenetics, and mutations is not always consistent with the disease phenotype. The differentiation of MDS with a high chance of leukemic transformation from other MDS also demands more information beyond mutations. Therefore, researchers used omics technologies to acquire downstream information to improve AML diagnosis.

### Omics in AML with FAB or WHO-defined aberrations

#### Transcriptomics

Several studies have applied omics technologies in profiling AML with recurrent genetic abnormalities. Virtaneva et al. [33]. profiled the expression patterns of 20 pretreated bone marrow (BM) samples of AML patients harboring isolated trisomy 8 (+ 8) or with normal karyotype (NK) using oligonucleotide-based DNA microarray. Compared with NK-AML, genes regulating apoptosis were significantly downregulated in with +8 abnormity, including the apoptosis inducer *BAD* and *CRADD*. However, *TP53* was increased in with +8 abnormity in response to the decrease of expression levels of apoptosis inducers *BAD* and *CRADD*. To determine whether gene expression profiling (GEP) can provide evidence for subtypes of AML with t(8;21)(q22;q22), t(15;17)(q22;q12), and inv [16](p13q22), Schoch et al. [34]. performed microarray analyses on BM samples from 37 AML patients with the above 3 cytogenetic aberrations. The three subtypes exhibited three distinct expression clusters across 1000 preselected genes, suggesting that genetically defined AML subgroups can be identified through GEP. Significantly lower expression of *X96719* was associated with AML with t(15;17) whereas significantly higher expression of *X96719* was associated with AML with t(8;21) or inv [16]. Studies by Debernardi et al. [35]. and Jiang et al. [36]. also reached the same conclusions concerning specific cytogenetics and expression patterns. In the Cancer Genome Atlas (TCGA) Research Network [37], information on RNA-seq in 179 AML samples and microRNA-seq in 194 samples was published along with related genomics and clinical data. The subtypes classified based on unsupervised clustering of RNA and miRNA expression were correlated with FAB subtypes and samples harboring certain mutations also displayed distinct mRNA and miRNA signatures.

Weighted gene co-expression network analysis (WGCNA) is a clustering method to identify gene modules associated with certain characteristics [38, 39]. Guo et al. [40]. used WGCNA to analyze the co-expression modules in AML patients in the BeatAML cohort and combined the co-expression modules with ELN stratification to determine the effects of GEP on the prognosis of AML. Three modules significantly correlated GEP with *NPM1*, *RUNX1*, and *TP53* mutations, which are primary factors in the diagnosis and risk stratification of AML. The top genes of *NPM1* mutation-related module included *MEIS1*, *HOXA5*, *HOXA3*, *HOXA7*, *HOXA6*, *HOXA10*, *HOXB3*, *HOXA9*, *PBX3*, *HOXB4, and etc.*, which mainly participated in positive transcriptional regulation, negative cell differentiation regulation, and *HOX* gene activation. Similarly, the module related to *RUNX1* mutation significantly enriched in the cytokine-mediated signaling pathway and hematopoietic cell lineage, while genes in the “light green” module were highly related to *TP53* mutation. Therefore, networks of gene expression are of great significance in assisting in the identification and diagnosis of AML subtypes, and the molecular subtypes are major indicators for treatment choice and prognosis of AML.

#### Proteomics

As early as 2004, Cui et al. [41]. obtained BM aspirates from 61 AML patients of different FAB types and analyzed them with MALDI-TOF-MS and ESI-MS/MS. They identified many proteins with differential expression levels between leukemic cells and normal cells and between cells from different FAB subtypes of AML. They also discovered seven specifically expressed proteins in M2 and M3 samples, including proteinase 3 and Azurocidin. In 2006, Balkhi et al. [42]. used BM samples of 42 AML patients for MALDI-TOF MS analysis and found the proteins and PTMs that were significantly different between various subtypes of AML. For instance, β-O-linked N-acetyl glucosamine of hnRNPH1 was unique to AML cases harboring 11q23 and acetylation of calreticulin was connected to t(8;21).

Kramer et al. [43]. performed proteomic and phosphoproteomic analyses for 44 AML BM samples with complete clinical and mutational data selected from the TCGA dataset. They identified several protein dysregulations connected to common mutations and fusions. For instance, samples harboring *IDH1* or *IDH2* mutations showed increased levels of 2-oxoglutarate–dependent histone demethylases KDM4A/B/C, although the mRNA levels of these genes were not elevated. Samples with *NPM1* mutation displayed an increased abundance of KPNA4 and KPNB1 (both belong to the nuclear importin family). *FLT3-TKD* mutant samples had increased phosphorylation in nine tyrosine residues compared with *FLT3* WT samples. AML samples with PML-RARA fusion exhibited a unique phosphorylation signature. Therefore, proteomics could present some different information from the transcriptomics, which may also help to identify the different characteristics of AML.

#### Metabolomics

Wang et al. [44]. employed NMR-based metabolomics in studying the serum metabolic alterations between AML patients and healthy donors and between different AML subtypes. Serum samples of 183 *de novo* AML patients and 232 matched healthy donors were profiled and AML cases showed higher levels of multiple metabolites including phenylalanine, tyrosine, N-acetyl-glycoprotein, citrate, mannose, and glucose. Moreover, different metabolic alterations were also detected between AML patients within different cytogenetic background. Compared with the cases with favorable cytogenetic characteristics, the intermediate cases showed significantly higher levels of several amino acids, myo-inositol, choline, lactate, and HDL (high-density lipoprotein) and lower levels of VLDL (very low-density lipoprotein) and LDL ( low-density lipoprotein). Their study demonstrated dysregulated metabolic pathways in the serum of AML patients from different cytogenetic risk groups, ensuring NMR-based metabolomic methods using serum samples as a reliable and less invasive approach to studying AML. To explore the lipid patterns in AML cases with different karyotypes, Stefanko et al. [45]. applied shotgun MS in profiling the lipidome of BM aspirates from 16 AML patients with normal karyotype, t(8;21) and inv [16]. Principal Component Analysis (PCA) revealed significant differences between the lipidome of the t(8;21) group and the other 2 groups, and further analyses indicated that sphingolipids and ceramides were most distinct between subtypes. Compared with AML-NK, increased levels of ceramide backbone-containing lipids (sphingomyelin (SM), ceramides (Cer), and GM3 ganglioside) were found in t(8;21) cases, indicating a shift to glycosphingolipid synthesis. Similarly, the results also demonstrated that many kinds of metabolites from AML with t(8;21) were involved in sphingolipid pathways, including decreased abundance of SM and increased ceramide synthesis. This study based on metabolomic analysis of lipidomes identified type-specific signatures in AML.

Apart from cytogenetic aberrations, mutations were also associated with different metabolomic features. *FLT3-ITD* mutation significantly upregulates mitochondrial hexokinase and promotes aerobic glycolysis dependence, which is known as the Warburg effect [46]. Stockard et al. [47]. collected plasma samples from 16 pediatric AML patients (8 with *FLT3-ITD* mutation and 8 had *WT FLT3-ITD*) and applied LC-MS for metabolic profiling. A total of 21 metabolites in plasma and 33 metabolites in leukemic cells were significantly differed from *FLT3-ITD* status, involving several important pathways like lysophospholipid metabolism and purine metabolism and biosynthesis. Mutant *IDH1* or *IDH2* leads to the conversion of α-ketoglutarate (α-KG) to oncometabolite 2-hydroxyglutarate (2-HG). Accumulation of 2-HG further results in epigenetic dysregulation and cellular differentiation blockage, thus assisting leukemogenesis [48, 49]. These correlations between druggable mutations and consequent metabolic alterations may help identify drug targets, study resistance to associated drugs, and find solutions to overcome the resistance. This issue will be discussed later.

These studies based on FAB or WHO-defined AML subtypes and mutations with known clinical significances suggested that transcriptomics, proteomics, and metabolomics strategies were capable of distinguishing AML with different cytogenetic and genetic abnormalities, demonstrating them as reliable methods in further AML diagnostics and studies.

### Omics in refining molecular subgroups of AML

Although mutations and cytogenetic features are predictive of disease prognosis, the real clinical outcomes are sometimes different [50]. Imprecise classification leads to improper choice of therapeutic strategies, this remains an intractable dilemma in current AML management, especially when over 45% of patients belong to the intermediate-risk category [51]. Different omics technologies have been implemented in refining molecular AML subgroups.

Identifying the characteristic patterns of gene activation and silencing, the so-called “expression signatures”, can distinguish subsets of AML patients [52, 53]. The fifth edition of the WHO classification of AML has renamed APL (acute promyelocytic leukemia) with t(15;17) as AML with PML::RARA fusion. However, the novel PML-independent RAR fusions such as RARG were not included [54]. Zhu et al. [55]. organized a global collaborative study of AML with RARG rearrangements based on samples collected from 29 different study groups. They applied unsupervised hierarchical clustering on the GEP of 201 samples (22 RARG-rearrangement samples, 66 PML-RARA samples, and 113 non-M3 samples). Results showed that 18 (81.8%) of the 22 RARG rearrangement samples clustered together, strongly indicating a new subtype. Gruber et al. [56]. performed RNA-seq in 14 samples derived from 14 pediatric acute megakaryoblastic leukemia (AMKL) patients and conducted a validation of BM samples from 34 pediatric AML patients. The CBFA2T3::GLIS2 fusion resulting from chromosome 16 inversion [inv [16](p13.3q24.3)] was found in 27% of the patients. The OS of patients with CBFA2T3::GLIS2 was significantly worse than those without this fusion. Their study defined an unfavorable prognostic subtype of pediatric AMKL characterized by the CBFA2T3::GLIS2 fusion [57]. They further analyzed RNA-seq and exome sequencing data of 99 AMKL patients and found that samples with different HOX loci and NUP98::KDM5A had distinct gene expression signatures and may represent distinct subgroups.

The above studies used the presence or expressional differences of a single gene or the existence of a certain fusion gene to refine the AML subgroup while subgroups defined by the whole GEP can better reflect the characteristics of individuals. Transcriptomics is now capable of comprehensively demonstrating the differences across different molecular groups through GEP. Bullinger et al. [58]. studied the gene expression levels in 166 AML adults using complementary-DNA microarrays. Through the unsupervised hierarchical cluster analysis of the 6283 differentially expressed genes across groups, they found that the expression profiles of samples from the t(15;17) group exhibited a highly correlated pattern while the t(8;21) and inv [16] samples were less well correlated. Their results suggested that the patients with the same cytogenetic abnormality could be further divided into subgroups based on GEP. In fact, a considerable number of studies have subdivided AML patients through transcriptomics, with or without taking mutations and cytogenetics into consideration. Cytogenetically normal AML accounts for 40–50% of all AML. These patients are heterogeneous and are generally classified into the intermediate group [6]. Based on unique patterns of gene expression after unsupervised hierarchical clustering and principal component analyses, Bullinger et al. [58]. differentiated NK-AML into two groups consisting of 133 genes, and prediction of prognosis based on these genes resulted in high accuracy. In the two prognostically relevant groups, group I overexpressed *GATA2*, *DNMT3A*, and DNMT3B. In group II, the genes related to granulocytes and monocytes, including vascular endothelial growth factor (*VEGF*), were significantly expressed. Because patients with the normal karyotype lack reliable markers for risk stratification and treatment selections, the findings of Bullinger et al. are instructive in subclassifying these patients. Several other studies [47, 59–62] based on GEP also identified molecular subgroups with or without correlation to mutations and karyotypes, which may guide better therapeutic selections.

Although further validations in independent cohorts are required, these studies demonstrated that RNA-seq was capable of classifying AML patients into groups more closely related to phenotype. The coexistence of the correlation and discrimination between transcriptome with genome and cytogenetics indicates a complex relationship between different biological levels as well as the requirement of involving downstream omics data in the AML study. However, most proteomics or metabolomics-related studies focus on the identification of prognostic markers and drug targets. We believe that biological subgroups of AML based on differences in proteins and metabolites will be more consistent with disease phenotypes although it is difficult to incorporate them in the diagnostic criteria.

### Omics in predicting MDS to AML transformation

As we mentioned in the introduction, around 30–40% of MDS patients (MDSs) progress to sAML and some aggressive MDSs are more likely to progress to sAML. Therefore, identifying these MDS patients and administrating proper treatment may improve their survival. Meanwhile, due to the poor survival of sAML, distinguishing sAML from *de novo* AML is also of clinical significance. Bejar et al. [62]. invented a pattern to identify the progression of sAML by comparing mutations between groups of different risks. They also found that prognostic classification methods such as the International Prognostic Scoring System (IPSS) and the Revised IPSS (IPSS-R) are poor predictors of leukemic transformation. Therefore, more methods are needed to help predicting the progress of MDS to sAML.

#### Transcriptomics

Early in 2004, Tsutsumi et al. [63]. applied microarray harboring oligonucleotides to distinguish *de novo* AML from sAML progressed from MDS. They compared the GEP in AML with multilineage dysplasia (AML-MLD) (*n* = 11), sAML (*n* = 11), therapy-related leukemia (TRL) (*n* = 2), and *de novo* AML without dysplasia (*n* = 15). They identified 56 genes that may be potential molecular biomarkers for differential diagnosis between sAML and AML-MLD and most of them were related to nuclear functions, including high mobility group nucleosomal binding protein 2 (*HMGN2*), high mobility group box 1 (*HMGB1*), and nucleosome assembly protein 1-like 1 (*NAP1L1*). Overexpression of platelet factor 4 (*PF4*) and some ubiquitination-related genes indicated the diagnosis of AML-MLD instead of sAML. Similarly, 28 genes that might distinguish *de novo* AML from sAML were also identified. Among them, high expression of lysosomal-associated multi-spanning membrane protein-5 (*LAPTM5*) might indicate the diagnosis of sAML. Vasikova et al. [64]. applied microarrays to analyze the GEP in CD34 + cells of 8 MDS patients and divided them into early MDS (*n* = 4) and advanced MDS (*n* = 4). They identified 286 differentially expressed genes between the two categories. Among them, 136 upregulated genes and 150 downregulated genes were found in early MDS whereas *ADAM8*, a gene belonging to *ADAM* (disintegrin and metalloproteinase domain-containing protein) gene family [65], was expressed highly in refractory anemia with excess of blasts-2 (RAEB-2) and sAML patients, indicating the association between ADAM genes and the progression of MDS. *BIRC5* was also confirmed to have a negative correlation with blast proportion, the conventional prognostic marker in MDS. Additionally, *MPL* proto-oncogene thrombopoietin receptor (*TPOR*), a member of the JAK-STAT signaling pathway, is highly expressed in sAML patients compared to the health cohort [66]. The expression levels of these genes mentioned above were tightly correlated with the stages of MDS and had potential prognostic significance.

Picou et al. [67]. examined the GEP of antioxidant-related enzymes using BM cells of 97 MDS/sAML patients and 25 healthy controls. They compared the redox metabolism between groups by quantifying reactive oxygen species (ROS) levels in BM cells and paid close attention to the 28 transcripts encoding for major enzymes involved in the antioxidant cellular response. MDS and sAML were found to have significant disturbances in redox metabolism, including decreased expression of antioxidant genes, which could be potential biomarkers for the diagnosis of sAML and disease monitoring of MDS. Shiozawa et al. [68]. applied RNA-seq in profiling BM samples of 100 MDS patients and classified patients into two subgroups based on the GEP: an immature progenitor (IMP) group and an erythroid megakaryocyte (EMK) group. Notably, the leukemic transformation was only present in the IMP group. The upregulation of cell signaling pathways such as *MAPK*, *NOTCH*, and *JAK-STAT* signaling pathways that took part in hematopoietic differentiation and stem cell self-renewal was found in the IMP group whereas some genes in pathways associated with DNA repair and metabolism were downregulated. Their study proved GEP as a potential reliable predictor of transformation from MDS to AML, improving the prognosis prediction and therapeutic choices of MDS and sAML. Some patients classified as MDS before are now diagnosed as AML regardless of BM blast according to the latest edition of guidelines (like patients with NPM1 mutation). Therefore, we believe that some of the patients who progress quickly from MDS to AML may also be diagnosed as AML in the first place. GEP can be important in identifying these patients by comparing the GEP of MDS, *de novo* AML, and sAML. The biomarkers identified in the above studies also have the potential to diagnose AML regardless of blast if further validations are conducted.

#### Proteomics

Pseudouridylation (Ψ) of transfer RNA-derived fragments (tRFs) is closely related to activities of hematopoietic stem cells, and the dysregulation of Ψ on a stem cell-enriched tRF type, which has a 5’ terminal oligo guanine (mTOG), is common in aggressive MDS subtypes [69]. However, the mechanism of how this post-transcriptional program eventually causes disease progression remained poorly understood. A recent study using adapted HDX-MS to analyze hematopoietic stem cells revealed that the binding site between mTOG-Ψ and polyadenylate-binding protein cytoplasmic 1 (PABPC1) was through the RNA-recognition motif (RRM) domain of PABPC1. A decrease of mTOG-Ψ’s binding to PABPC1 results in an increase of PABPC1-interacting proteins 1 (PAIP1)’s binding to the same site on PABPC1. PAIP1 is a translational co-activator and its upregulation aberrantly increases the translation of its associated mRNA, which is involved in the MDS-to-AML progression [70].

A study conducted with TOF-MS identified the decreased levels of CXC chemokine ligands 4 (CXCL4) and 7 (CXCL7) in PB samples of advanced MDS. These two proteins may be potential markers in predicting MDS progression [71]. More recently, researchers found a downregulation of the protein FBXO11 in sAML patients. They applied LC–MS/MS to identify the ubiquitin substrates of an FBXO11-associated E3 ligase in the MDS cell model. Those decreased ubiquitinated peptides in *FBXO11*-knockout cells were analyzed and turned out to be involved in the processing and metabolism of RNA. Therefore, they suggested that FBXO11 was a potential marker in predicting the leukemic transformation of MDS [72]. Another study acquired BM and PB samples from *de novo* AML patients and MDS patients before transforming to AML and used MALDI-TOF MS to analyze and compare their proteome profiles. The authors found 3 significantly upregulated proteins, including moesin, ezrin, and apoptosis-inducing factor mitochondria associated 1 (AIFM1) in *de novo* AML, which may be biomarkers in distinguishing *de novo* AML from sAML [73].

However, limited proteomics-based studies have been conducted in studying the transformation from MDS to AML and some of biomarkers also lack further verification. As we discussed above, some MDS patients should be diagnosed as AML in the first place and proteome information will be important in identifying this group of patients.

#### Multi-omics

Murine double minute X (MDMX) is a suppressor of p53 [74]. It has been reported that MDMX is overexpressed in about 90% of AML patients, the functional consequences of this overexpression remain unclear [75]. Researchers integrated RNA sequencing and LC-MS/MS in studying preleukemic BM cells and found that MDMX overexpression caused the transition from preleukemic stem cells to leukemic cells through upregulating the Wnt/β-catenin signaling pathway. As MDS RAEB patients tend to have elevated MDMX and a higher chance of transforming to AML, they considered it a marker indicating the progression from MDS to AML [76].

### Omics studies in the prognosis of AML

Although it is relatively easy to diagnose AML based on morphological abnormality, precise risk stratification for the diagnosis of AML is difficult. As we discussed above, the risk stratification by ELN based on genetic and cytogenetic information is sometimes inconsistent with the actual clinical prognosis, demanding more information to be integrated into the risk-stratifying criteria. Therefore, a considerable number of studies implemented omics approaches aiming at refining the risk stratification of AML.

### Transcriptomics

The expression level of a single gene, several related genes, the presence of fusion genes, and the whole expression profile can all be used as markers for prognosis prediction and for AML subgroup classification.

Early in 1999, Golub et al. [77]. reported that the upregulation of *HOXA9* was related to the poor outcome of AML based on gene expression monitored by microarrays. Andreeff et al. [78]. extended the finding of Golub et al. and studied the expression of *HOX*, *FLT3*, and *MLL* genes in 199 patients with newly diagnosed AML. They found that the downregulation of *HOX* expression was a consistent characteristic of AML with a favorable prognosis and the lower level of *HOXA9* expression was the best predictor of overall survival (OS) and disease-free survival (DFS). HSPG2, synthesized by BM cells, plays an important role in hematopoietic cell differentiation but is still mysterious in the mechanism of AML [79]. Zhou et al. [80]. applied RNA-seq in the BM mononuclear cells collected from 4 AML patients and 3 healthy controls to analyze the association between *HSPG2* expression and the clinical outcomes of AML patients. The results showed that *HSPG2* was significantly upregulated in AML patients than in healthy controls and the *HSPG2* expression decreased in the complete remission (CR) phase but increased after relapse. AML patients with high expression of *HSPG2* were more likely to have shorter OS and leukemia-free survival (LFS). Considering the results mentioned above, *HSPG2* may be a pro-oncogene in AML pathogenesis with the potential to be a predictive factor for poorer prognosis. Bottomly et al. [81]. combined genomics data, transcriptomics data, and clinical outcomes to determine the functional genes that can help predict the prognosis of AML. They highlighted the impact of AML LSCs in the disease pathogenesis and relapse and found a single targetable gene that can determine the overall survival in AML. Platelet endothelial aggregation receptor 1 (*PEAR1*) was associated with an HSC-like signature and its expression level can predict the poor AML prognosis irrespective of ELN classification. The prognostic marker independent of ELN risk stratifications indicates that transcriptomics enables the refinement of AML subgroups from a different aspect.

### Proteomics

In 2008, Forshed et al. [82]. demonstrated a workflow for identifying AML protein biomarkers based on SELDI-MS data. From then on, as proteomic techniques progress rapidly, an increasing number of studies have been carried out using these techniques in discovering prognosis biomarkers of AML.

Friend leukemia virus integration 1 (FLI1), a member of the ETS transcription factor family, is involved in normal hematopoiesis and its overexpression was associated with the progression of some solid tumors and hematological diseases [83–85]. To study the role of FLI1 in AML, Kornblau et al. [86]. performed proteomics profiling in PB and BM samples of 511 AML patients at diagnosis with reverse-phase protein array, the expression of FLI1 as well as 195 other proteins was measured. The FLI1 expression was higher in 31.8% and lower in 4.8% of samples from AML patients compared to normal CD34^+^ cells. Among the other 195 proteins, 10 proliferation and stromal interaction-associated proteins were negatively correlated with FLI1 levels. They further found that patients with high and low levels of FLI1 both had shorter duration of remission. High expression of FLI1 was a risk factor for adverse prognosis. However, the predictive value of FLI1 was not validated in other cohorts.

A study based on SELDI-TOF MS subdivided the intermediate and unfavorable-risk AML groups according to proteome profiles [87]. They performed protein profiling on BM and PB samples of 54 *de novo* AML patients before treatment and divided them into two proteomic clusters with significantly different overall and event-free survival rates. Additionally, patients belonging to the intermediate-risk group were split into two proteomic groups, one had a similar prognosis as the favorable-risk group and the other had a similar prognosis as the unfavorable-risk group. They further verified S100A8 as a marker for poor prognosis with a specificity of 75% and a sensitivity of 70% for death prediction. Notably, previously mentioned transcriptomics-based studies found elevated expression of *S100A8* gene in samples treated with FLT3 inhibitors and its level might be associated with resistance to FLT3 inhibitors [88]. Higher expression of *S100A8* gene was also related to poor prognosis and chemotherapy resistance in *de novo* AML [89]. The consistency of expression level between different omics layers was observed for *S100A8*, which makes it a more reliable marker for poor prognosis.

Around 20% of the mutations in AML were associated with RNA splicing and chromatin modification [90]. Mutated epigenetic modifiers combined with other mutations result in prognostically distinct subtypes, thus complicating individual prognosis stratification and treatment selection [91–93]. Epigenetic modifications of histone modifiers have been studied as dysregulations in some solid tumors [94–96]. Djik et al. [97]. applied proteomics profiling in studying prognosis-associated epigenetic modifications on histone modifiers in AML. H3K4me2, H3K4me3, and H3K27me3 modification levels were examined in 241 samples from AML patients and 188 acute lymphoblastic leukemia (ALL) patients. H3K4me2, H3K4me3, and H3K27me3 levels were significantly lower in AML samples than in normal or ALL samples. In both univariate and multivariate analyses, greater reduction of H3K27me3 was associated with shorter overall survival, for all AML patients and AML cases with DNA methylation mutations or *TP53* mutation. Although further validations to calculate the sensitivity and specificity are needed, their study proved that proteomic profiling of histone methylation was a reasonable approach to identifying prognosis biomarkers for AML with different mutations.

Zhang et al. [98]. obtained serum samples from 51 AML patients within different risk groups (14 with favorable risk, 19 with intermediate risk, and 18 with adverse risk) and profiled their serum proteome based on TMT (tandem mass tag)-MS/MS. A total of 138 differentially expressed proteins were identified between groups and among them, elevated levels of FH, IDH2, ENO1, LTF, and GLUL were significantly associated with poor prognosis. ELISA assay confirmed their upregulation discovered by MS. They considered these proteins to be potential biomarkers for AML with poor prognosis. However, their study was based on cytogenetically defined risk groups, while protein biomarkers which can predict outcomes irrespective of ELN risk stratifications may have more clinical significance.

Zhang et al. [99]. used label-free quantitative proteomics in profiling the proteome of 10 BM plasma samples of newly diagnosed AML and 3 healthy donors. They identified the differentially expressed proteins (DEPs) in AML samples and further studied the correlation between DEPs and survival data. The survival of patients within ELN-2017 intermediate-risk group with high intercellular adhesion molecule-2 (ICAM2) expression was very similar to those in the adverse-risk group. Therefore, upregulated ICAM2 protein in BM plasma was a predictive factor for the survival in the intermediate-risk subgroup. A high proportion of AML patients belong to the intermediate-risk group and treatment decision is difficult in choosing HSCT (like the adverse-risk group) or chemotherapy (like the favorable-risk group) after the first CR [100, 101]. HSCT may be performed for AML patients harboring elevated protein level of ICAM for these patients exhibit poor prognosis similar to the adverse-risk group.

### Metabolomics

An MS-based metabolomics study conducted by Chen et al. [102]. on PB samples from 400 AML patients and 446 healthy donors identified the glucose metabolism signature in AML. Six metabolites (lactate, 2-Oxoglutarate, 2-HG, pyruvate, glycerol-3-phosphate, and citrate) were differentially expressed in AML samples in both training and validation cohorts and were all associated with glucose metabolism. They then incorporated the six identified metabolite biomarkers into a prognosis risk score for predicting patient survival. The score was independently predictive of patients with worse prognosis in the absence of well-established predicting markers, indicating an AML subgroup with unfavorable prognosis based on glycolysis metabolism. Based on this study, they speculated that leukemic cells increased fructose utilization by upregulating GLUT5 (a fructose transporter) to compensate for glucose insufficiency [103]. They then observed increased fructose uptake and elevated expression of GLUT5 in leukemic cells, as well as decreased serum fructose in the samples of AML patients. Furthermore, they adapted a multivariate Cox model and found that higher SLC2A5 expression was also significantly linked to poor overall survival. Their two studies based on metabolomics approaches successfully identified reliable serum prognostic markers for AML patients. Pabst et al. [104]. applied several MS methods to comprehensively determine the serum lipid profile of 20 *de novo* AML patients. Elevated levels of arachidonic acid (ARA) and its precursors were associated with unfavorable prognostic risk, as well as with higher blasts in PB and BM than cases harboring lower level of ARA. Meanwhile, an increased level of prostaglandin F2α (PGF2a) was related to lower peripheral blasts and favorable prognostic risk, indicating a potential marker for a subgroup with a better prognosis.

Because examining the metabolites from serum samples is a less invasive approach, constructing a metabolomic-based method for risk stratification may be promoted to large-scale use in clinical practices. However, metabolites may change rapidly due to slight interference, making it difficult to find reliable markers.

### Multi-omics

We have summarized studies implementing single omics approaches in identifying prognostic biomarkers. We believed that integrating data from different omics layers would provide valuable markers. However, probably because markers identified by multi-omics approaches are more reliable than using single-omics approaches, most of the markers were further verified as potential drug targets. Therefore, prognostic markers in multi-omics studies will be discussed later in the section on targeted therapy.

## Omics in AML treatment

### Omics in studying chemotherapy response of AML

Being the fundamental treatment of AML, chemotherapy remains the therapeutic strategy for most patients. Many AML patients fail to achieve CR after induction chemotherapy or relapse soon after remission. Therefore, it is of significance to reveal the molecular mechanism for chemotherapy resistance and to find reliable markers to predict response [105].

#### Transcriptomics

It is known that MLL-AF9 (KMT2A::MLLT3) and NUP98-NSD1 were closely related to the chemotherapy resistance and resulted in high rates of relapse [106–108]. Apart from these fusion genes associated with treatment response, transcriptome can reflect the cell state after treatment and monitor minimal residual disease (MRD) after chemotherapy. It was also applied to detect changes during therapy and analyze the potential causes for chemotherapy resistance and relapse.

Heuser et al. [109]. found high expression of MN1, FHL1, CD34, RBPMS, LPAR6, and NPR3 genes was related to chemotherapy resistance based on cDNA microarrays. Moreover, AML patients with *NUP98-NSD1* fusion or *NUP98* rearrangement were resistant to chemotherapy [107, 110]. Floren et al. [111]. applied RNA-seq to identify the increased expression of *CD82*, a membrane scaffold reported to be associated with leukemia cells [112], in AML patients after standard chemotherapy. They further indicated a strong correlation between the overexpression of CD82 and poor treatment response as well as worse prognosis in pediatric AML patients by using the Therapeutically Applicable Research to Generate Effective Treatment (TARGET) AML database.

Zhai et al. [30]. compared the gene expression differences between diagnosis (Dx) and relapse (Re) pairs of AML patients at a single-cell level and observed a significant clonal expansion and evolution in the progression of AML. Gene fusion and mutation detection based on RNA-seq showed that *KIT* mutation increased the risk of poor prognosis and recurrence [113]. The expression changes in six AML pairs showed that differentially expressed genes like *LOXL1* and *FAM81A* were more likely to appear in the relapse (Re) group. In *FLT3-ITD* patients, compared with the Dx group, Re patients had decreased expression of *AP-1*/*ATF-2* and increased expression of *mTORC1* targets, which revealed a pathway shift from *AP-1* to *mTORC1*. At the same time, the upstream *KRAS* gene was also upregulated in relapse patients. These studies explored the molecular mechanism of chemotherapy resistance and relapse patients, which can provide new insights into therapeutic strategies for AML patients [114].

Several studies reported that AML-initiating cells (LICs) can evade chemotherapy-induced cell death and promote disease progression and relapse [115–117]. To explore the role of LICs in chemotherapy-resistant and relapse AML patients, Stetson et al. [118]. performed scRNA-sequencing on 813 LICs from 5 matched samples from AML patients at diagnosis and after relapse. Twenty-two marker genes that differentially expressed in RNA clusters were defined as AML LICs membership and *KLF6*, *ENO1*, *TPI1*, and *TALD01* were found to be significantly downregulated in relapse groups, whereas *CD44*, *HLAs*, and *PTMA* were highly expressed in relapse groups. Dominant gene expression cluster at relapse was enriched for pathways including TNFα and IL6/JAK/SAT3, which were potentially therapeutically targetable.

The BM tumor microenvironment (TME), which not only facilitates the growth of leukemic cells but also initiates leukemogenesis of healthy cells, is important in disease progression [119]. Mumme et al. [120]. utilized scRNA-seq to analyze BM samples in four pairs of AML patients at Dx, at the end of induction (EOI), and after relapse, and obtained a blast cell-related seven-gene signature associated with relapse and survival, including *CLEC11A* (a growth factor for hematopoietic progenitor cells [121]), *PRAME* (a RAS target promotor inhibiting the differentiation and apoptosis induced by retinoic acid [122]), *AZU1* (a myeloid differentiation factor), *NREP*, *ARMH1*, *C1QBP*, and *TRH*. The expression of the genes mentioned above, except *TRH*, was high in Dx AML blast cell clusters compared with non-blast cell clusters from both Dx and EOI time points, indicating an AML blast signature. Survival analysis proved the significant correlation between the high expression level of the 7-gene signature and poorer OS. The longitudinal analyses of samples in Dx, EOI, and relapse demonstrated that the blast cells were identified in Dx, reduced in EOI, and reappeared in relapse. TME exhibited its significance in pediatric AML relapse or continuous CR. Zhang et al. [123]. applied scRNA-seq in BM samples from 13 pediatric AML patients before and after chemotherapy. Cells were clustered based on GEP and about 50% of leukemic stem/progenitor cells exhibited LSC and oxidative phosphorylation (OXPHOS) signatures. Cells from these clusters had strengthened metabolic programs and were associated with chemoresistance. Importantly, *CD69* was highly expressed in chemoresistant LSC-like subpopulations, which may be the marker for identifying chemoresistant LSCs. The scRNA-seq-based studies comprehensively characterized the TME of AML and provided valuable information on treatment response and prognosis prediction.

Several above studies identified dysregulated expression of genes related to cell differentiation, including *CD82*, *CD53*, and *CD69*. The markers were consistent with the clinically used markers for MRD, which was conducted by flow cytometry. Therefore, further studies combining the expression of these genes and flow cytometry may improve the monitoring accuracy. Elevated expressed genes after chemotherapy may be the potential targets for overcoming chemoresistance. Further studying the protein levels of these chemoresistance-related genes at the ex vivo and in vitro levels is needed.

#### Proteomics

In 2009, Albitar et al. [124]. implemented SELDI and a Ciphergen ProteinChip system to profile proteins in PB samples from 41 AML patients with intermediate or poor cytogenetics before the cytotoxic therapy. Seventeen patients responded well while 24 patients did not achieve CR. MS peaks correlated with treatment response were selected and those with the highest correlation were used to construct a prediction model in combination with other characteristics including age, blasts, and cytogenetics. The model was able to predict responders with an accuracy of 95% and non-responders with an accuracy of 85%. Later, Kaźmierczak et al. [125]. obtained the PB and BM samples from 30 AML patients before treatment and 17 samples from healthy donors, the patients later received standard “7 + 3” induction therapy. Among the patients, 18 achieved CR, 7 were resistant to induction therapy and 10 patients relapsed. They performed ESI-MS/MS to compare the proteome of the samples from patients with different treatment responses. There was no statistical difference in the protein expression between samples before and after relapse, while differences were observed between those who achieved CR or were resistant to therapy. Four proteins, annexin I, glutathione transferase ω, esterase D, and γ1 actin, were significantly correlated with treatment response. Annexin I was significantly upregulated in patients who achieved CR and γ1 actin was upregulated in patients resistant to induction chemotherapy. The other two proteins, esterase D and glutathione transferase ω, were exclusively detected in patients with CR, which were the best predictors of CR (*P* = 0.0032).

Aiming at finding therapeutic solutions for chemoresistant patients, Zhu et al. [126]. profiled the phosphoproteome in primary cells derived from 8 patients at diagnosis. These patients later received “7 + 3” induction therapy and 4 reached CR while 4 exhibited treatment failure. The phosphoproteomics data demonstrated increased phosphorylation in proteins associated with FLT3, MAPK, and ATM signaling in refractory cases. NetworKIN analyses of upregulated phosphoproteins showed that refractory cases had increased phosphorylation in putative substrates of CK2 and CDK family. Further KEA2 analysis showed that the activity of CK2 and CDKs was also higher in the refractory samples. Consistently, the CK2 inhibitor CX-4945 could significantly increase cytarabine-induced cell death in cells from the refractory samples. Rosales et al. [127]. profiled the phosphoproteome in AML cell lines treated with CX-4945 and observed the influence of the CK2 inhibitor CX-4945 on important pathways and biological processes associated with chemosensitivity and survival of cells. Therefore, CK2 may be a potential target in combination with chemotherapy. Zhu et al. also found that HMGA1, a CK2 substrate associated with chemoresistance in lung cancer [128], was one of the significantly phosphorylated proteins detected in refractory samples. Knockdown of *HMGA1* in AML cell lines markedly decreased cell proliferation. In contrast, the colony formation was increased in an MLL-AF9/*FLT3-ITD* murine model with a mutant form of HMGA1 mimicking constitutive phosphorylation by CK2. Their study revealed that HMGA1 phosphorylation by CK2 could promote resistance to cytarabine and blocking HMGA1 phosphorylation using CK2 inhibitors sensitized these chemoresistant AML cells.

Although CR is achieved, some patients still experience early relapse and monitoring of MRD after CR is crucial. Research predicted that the proteome of leukemic blasts would be different from normal blasts and the distinct proteome might be potential biomarkers in confirming CR status [129]. A study based on 2-DE and MALDI-TOF MS mapped the proteome of mononuclear cells from the BM of 12 AML patients and 2 normal volunteers [130]. Compared with non-remission patients, the B-cell translocation gene 1 (BTG1) protein was upregulated in the BM mononuclear cells of CR patients (AML-M2 and M3) and healthy volunteers. Thus, BTG1 level may be a treatment-related biomarker in monitoring CR status of AML-M2 and M3. Aasebo et al. [131]. utilized LC-MS/MS to profile the proteome and phosphoproteome of blast cells derived from 41 AML patients at diagnosis. Note that all patients later reached CR. Protein expression or phosphorylation was different between patients who relapsed within 5 years and patients who did not. Increased expression of RNA processing proteins and increased phosphorylation of CDKs and CK2 were found in relapsed cases, whereas relapse-free ones exhibited increased levels of V-ATPase proteins. Adding to the study mentioned above by Zhu et al. [126]. , CK2 phosphorylation may be associated with both chemoresistance and relapse, making it a promising drug target.

Although many proteome-related factors are associated with response to chemotherapy and some of them are predictive of the response, further studies on overcoming chemoresistance based on the detected mechanisms are needed.

#### Metabolomics

The extremely flexible and diverse metabolism leads to the disease aggressiveness as well as the tendency to drug resistance in AML [132]. Targeting metabolic disorders and monitoring clinical responses may be utilized in personalized medicine [133]. Previous studies have demonstrated that AML cells, like other cancer cells, are capable of undergoing compensatory metabolic adaptations in response to the chemotherapies or drugs targeting certain pathways, adding difficulties to sustainable treatment strategies [134, 135]. Relapses after tumor regrowth initiated by chemoresistant leukemic clones after chemotherapy contributed to the poor prognosis [136]. Metabolomics has been used in exploring solutions to chemoresistance in some studies.

In a previously mentioned study by Chen et al. [102]. in which a prognostic system of 6 glycolysis related metabolites was developed, they further demonstrated in an in vitro study that a high level of glycolysis contributed to refractory to arabinofuranosyl cytidine (Ara-C) while inhibition of glycolysis strengthened the cytotoxicity induced by Ara-C and suppressed AML cell proliferation. Therefore, inhibition of the glycolysis pathway may be a potential therapeutic target for reinforcing the effects of chemotherapy in AML. Stockard et al. [137]. performed LC-MS-based global and targeted metabolomics on 94 serum samples from pediatric AML patients later treated with cytarabine. A few metabolites were found significantly associated with treatment response and survival. Among them, a higher abundance of pantothenic acid was connected to poorer half-maximal inhibitory concentration (IC_50_) and relapse-free survival (RFS) outcomes, indicating a relationship between uptake of pantothenic acid and cytarabine resistance. In terms of the metabolic pathway, amino acid synthesis-related pathways (including the metabolism of aspartate, glutamate, and pantothenic acid-based CoA biosynthesis) were significantly associated with IC_50_ and RFS. Their results were consistent with an earlier study conducted by Stäubert et al. [138]. in which the global untargeted metabolomics discovered that chemoresistant leukemia cells were characterized by decreased glutamine dependence, reduced uptake of pantothenic acid, and dysregulated fatty acid β-oxidation. Stockard et al. [139]. profiled the metabolome of 7 cell lines with different sensitivity to cytarabine and doxorubicin through ultra-high-performance LC-MS. Metabolites with significant differences between cell lines sensitive or resistant to cytarabine included D-raffinose, guanosine, inosine, guanine, aldopentose, allopurino, 4-hydroxy-L-phenylglycine, and glucosamine/mannosamine. Pathway analyses showed that disturbance in purine metabolism was associated with resistance to cytarabine. For cytarabine, levels of several amino acids were distinctive between sensitive and resistant cells, indicating the participation of amino acid metabolism in resistance to cytarabine.

The metabolites and metabolic pathways identified in the above studies were associated with chemosensitivity, which may serve as biomarkers in predicting drug response and indicate potential drug targets.

#### Multi-omics

Brown et al. [140]. performed RNA-seq on AML specimens from patients who responded well and who were resistant to induction chemotherapy. They reported the overexpression of *MEF2C* in relapse and chemotherapy-resistant AML patients. It was previously reported that high *MEF2C* expression in chemoresistant pediatric AML with adverse prognosis can be used as a response biomarker [141]. Phosphoproteome analyses discovered significantly elevated levels of MEF2C phosphorylation at S222. They then established *MEF2C* mutation knock-in mice model and found phosphorylated MEF2C to be important in primary chemotherapy resistance through maintaining LSCs. MEF2C phosphorylation at S222 can enhance the transcriptional activity and induce apoptosis and therefore, is a distinct marker for chemoresistance. A combination of transcriptomic and phosphoproteomic assays could identify differentially expressed genes as well as the PTM, phosphorylation, identifying more specific biomarkers for the prediction of chemoresistance.

### Omics in studying targeted therapy of AML

Generally, among multiple mutations detected in AML patients, only very few of them are therapeutic targets like *FLT3* and *IDH1*/*IDH2* [142–144]. Unfortunately, poor response, resistance to these inhibitors, and early relapse are frequently occurring phenomena in clinical practice, and the mechanisms behind the resistance need further studies. Therefore, some transcriptomics-based studies were conducted to elucidate the mechanisms of how the resistance occurs to targeted drugs and to explore therapeutic solutions to overcome the resistance. New drug targets are also being discovered using transcriptomics approaches. We hereby summarize current omics-based studies on targeted therapies that have been approved for clinical use or potential targets that are still under investigation.

#### Omics studies in AML patients harboring FLT3 mutation

**Fig. 4:**
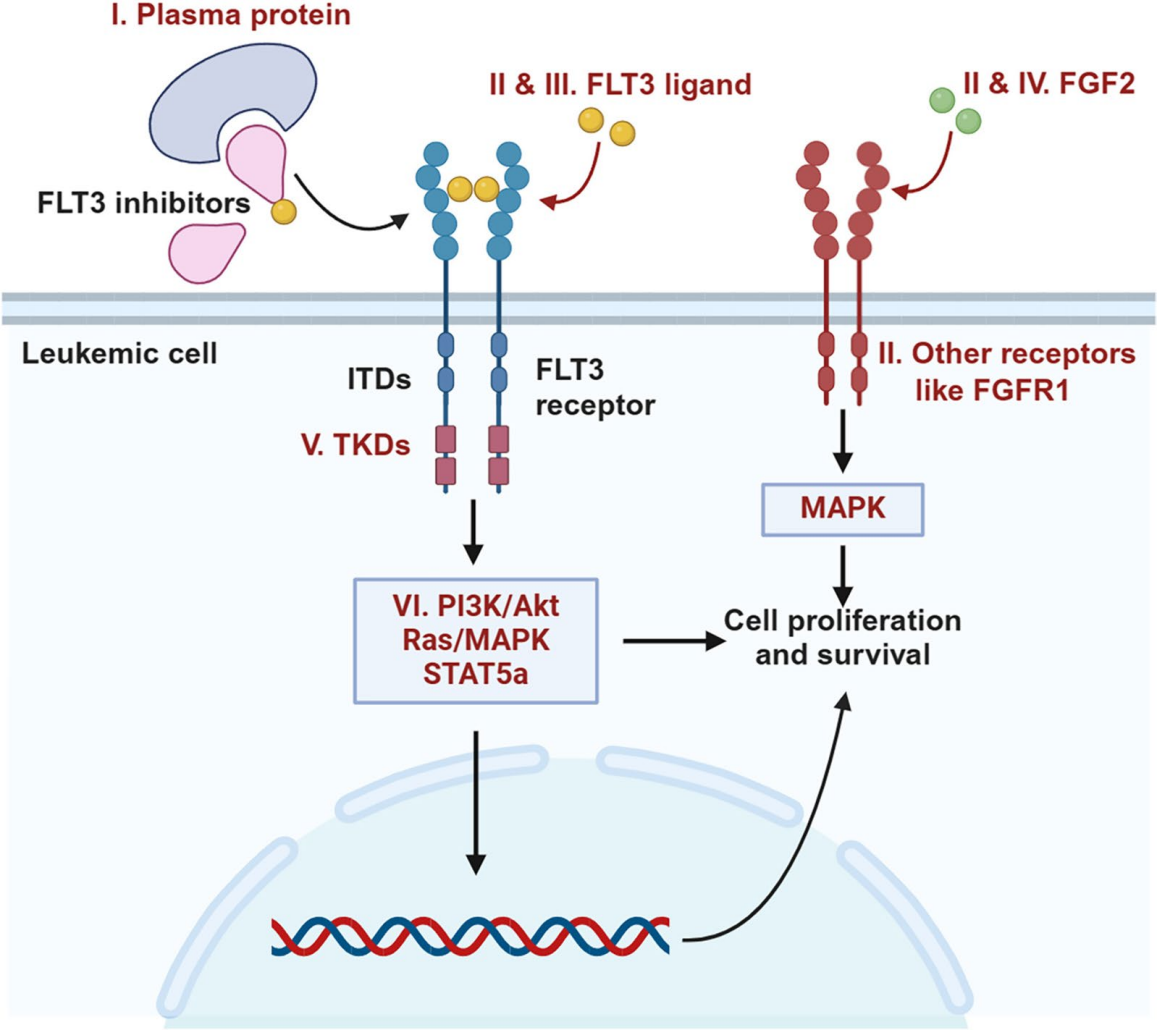
Molecular mechanisms for the resistance of AML to FLT3 inhibitors. The mechanisms associated with the resistance to FLT3 inhibitors are in red. (I) The binding of plasma proteins (like acid-glycoprotein) to FLT3 inhibitors can impair the efficacy of FLT3 inhibitors. (II) FLT3 ligand and FGF2 are extrinsic microenvironmental proteins which prevent FLT3-ITD (+) cells from apoptosis induced by FLT3 inhibitors. (III) FLT3 ligand competes with the inhibitors for receptor binding. (IV) FGF2 binds with other receptors like FGFR1 and activates MAPK signaling and therefore induces leukemic cell proliferation. (V) Acquired TKD (tyrosine kinase domain) mutations prevent the binding of FLT3 inhibitors to receptors as well as activating downstream signaling pathways without upstream signals. (VI) Dysregulation of PI3K/AKT, RAS/MAPK, and STAT5a signaling pathways with abnormal protein levels or phosphorylation results in leukemic cell proliferation

 The general mechanisms of resistance to FLT3 inhibitors include abnormal stimulated pathways, competing ligands, and upregulation of antiapoptotic proteins (Fig. 4) [145]. The mechanism of signaling pathway mutations downstream of FLT3 like RAS mutations promises phosphoproteomics approaches as an important tool for studying resistance to FLT3 inhibitors [146, 147]. Although limited solutions have been brought up to overcome the resistance or simply to predict resistance, a number of studies based on omics approaches have been conducted in this field.

##### Transcriptomics

Stölzel et al. [148]. used microarrays to profile the gene expression of midostaurin-resistant or sensitive AML cell lines with *FLT3*-mutation. They found new aberrations like the upregulated antiapoptotic genes and downregulated proapoptotic signals which might contribute to the chemotherapy resistance. Kivioja et al. [149]. performed NGS in analyzing RNA and exome of BM samples from 87 AML patients (38 with *FLT-3 ITD* mutation) and 13 healthy donors. Analysis of the relationship between gene expression and response to sorafenib revealed that patients with high *HIF* expression had better responses. Zavorka et al. [88]. applied transcriptomic analyses to identify the alternative mechanisms of gilteritinib resistance based on *FLT3-ITD* mutant murine models. By comparing the GEP before and after gilteritinib administration, they found that 25 genes were significantly upregulated after gilteritinib treatment, including *S100A8* and *S100A9*. Further studies based on a transcription factor screen identified *BCL6* (a transcriptional corepressor) as the regulator that could upregulate *S100A9* expression under the effect of gilteritinib. Inhibiting *BCL6* could promote the growth of AML cells with *FLT3-ITD* mutation and resistance to gilteritinib. Their findings suggested a new mechanism of giltertinib resistance and identified a potential therapeutic target to overcome gilteritinib resistance. For AML patients harboring both *FLT3-ITD* and *NPM1* mutations, the combined use of FLT3 inhibitor and menin inhibitor was proposed [150]. Dzama et al. [151]. combined a novel menin-MLL inhibitor VTP-50,469 with FLT3 inhibitor quizartinib to treat human and murine leukemic cells with *NPM1* mutation or MLL rearrangement and performed RNA sequencing on cells after both combined treatment and monotherapy. Gene set enrichment analysis showed that the genes downregulated after the cells were treated with either menin-MLL inhibitor or FLT3 inhibitor were significantly enriched for *STAT5A* (an *FLT3*-activated transcription factor) target genes, and the combined treatment yielded a more significant downregulation in genes downstream of FLT3 signaling. The combined treatment also exhibited better efficacy for AML cells with *NPM1* and *FLT3-ITD* mutation. Their study proposed a treatment strategy of the combination of menin and FLT3 inhibitors for AML patients with *NPM1* mutation and MLL rearrangement and concurrent mutation of *FLT3-ITD*. Because harboring both *NPM1* and *FLT3-ITD* mutations is relatively common in AML, the combined treatment is highly valuable. Further trials are needed for the combination of these two drugs with chemotherapy or azacitidine.

##### Proteomics

So far, several studies have applied proteomics approaches in predicting FLT3-TKI response of AML patients. Roolf et al. [152]. identified different pathways inhibited by sorafenib in *FLT3-ITD* (+) and (-) cells based on phosphoproteome analyses. Inhibition of *FLT3-ITD* (-) cells was achieved by suppressing MEK/ERK signaling. Their result suggested the presence of determinants other than *FLT3-ITD* for the treatment response of sorafenib. Cucchi et al. [153]. performed phosphoproteomics using 35 AML samples, 17 *FLT3*-mutated and 18 *FLT3*-wild type (WT), in combination with studying ex vivo response to identify differential phosphorylation correlated to response to FLT3 inhibitors (gilteritinib and midostaurin). Because responses were also observed in *FLT3*-WT samples, they then studied phosphoproteomic profiles independent of *FLT3* mutation status. They found that samples resistant to gilteritinib had increased phosphorylation of MAPK, KIT, and FGFR1, suggesting that these alternative pathways contribute to drug resistance independent of FLT3. The conclusions were consistent with those of another study conducted by Casado et al. [154]. , in which they combined untargeted MS-based proteomics and phosphoproteomics in cells from 36 AML patients and found mutations in RAS signaling were associated with resistance to FLT3 inhibitors. Schaab et al. [155]. combined super-SILAC with quantitative MS in patient-derived AML blasts to identify phosphorylation sites as predictive biomarkers for quizartinib treatment. A total of 5 sites were selected and further verified as predictive markers for quizartinib treatment in *FLT3-ITD* mutant patients. The sites identified were S160 in EEPD1, S630 in BCL11A, S333 in RANBP3, S961 in RP3, and S458 in LMN1. In 2022, Koschade et al. [156]. applied functional translatome proteomics with phosphoproteomics in studying cellular responses to FLT3 inhibitors in *FLT3-ITD* mutated AML. They found autophagy through AKT-mTORC1-ULK1 to be a crucial mechanism for primary resistance of FLT3 inhibitors. Hijazi et al. [157]. used kinase substrate enrichment analysis (KSEA) algorithm to predict drug response of trametinib, midostaurin, and silmitasertib based on proteomic and phosphoproteomic data. The models predicted drug response with a relatively high accuracy (20–40%). The above studies based on proteomics and phosphoproteomics approaches highlighted the participation of MEK/ERK and AKT signaling pathways in the development of resistance to FLT3 inhibitors. Not only did they facilitate the prediction of treatment response to FLT3 inhibitors, but also they provided clues for drug targets in combination with FLT3 inhibitors.

##### Multi-omics

A study conducted by Gosline et al. [158]. demonstrated the proteome and phosphoproteome profile in 38 AML cases with available genomics and transcriptomics data. Selected features from different omic layers were tested, both separately and combined, for their capability of modeling ex vivo responses to a total of 26 drugs. Gene mutations were inaccurate in modeling responses to targeted therapies like *FLT3* mutation for quizartinib and *NRAS* mutation for trametinib, but models including mRNA and protein features exhibited better performance in modeling drug responses. The proteins and phosphopeptides selected for predictive models of quizartinib and trametinib could cluster AML cells on the basis of response to these two drugs. Patients with poor response to trametinib highly expressed some proteins linked to mRNA processing and catabolism. Prize Collecting Steiner Forest (PCSF) algorithm based on mRNA and protein data was applied in network integration. Numerous apoptotic associated proteins like BID, CASP1, and GZMB were identified, indicating that levels of apoptotic-related proteins and transcripts could affect sensitivity to trametinib. Although mutations could not correctly predict response to quizartinib and trametinib, addition of mRNA expressions and protein levels into the model improves the accuracy of sensitivity prediction.

Attempting to demonstrate the course of gilteritinib resistance in AML patients harboring *FLT3-ITD* mutation, Joshi et al. [159]. performed a comprehensive multi-omics analysis including genomics, proteomics, and metabolomics using AML cell lines and patient samples. Results showed great differences in proteome and metabolome profiles between samples from the early resistance and late resistance groups. Early-stage resistance was complex including dysregulation in lipid metabolism and PI3K/MAPK signaling pathway. An aurora kinase B (AURKB) dependent cell cycle progression was also observed in early resistance. Late resistance was dominated by *NRAS* mutations and continued metabolic reprogramming including a more prominent dependency on phospholipid metabolism in FGF2. Inhibition of AURKB could resensitize early resistance to gileritinib in AML cells. As gaining *NRAS* mutation would cause resistance [160], early application of the FLT3 inhibitor as well as the AURKB inhibitor may stop the progression of resistance in the early stage before *NRAS* mutation occurs. Compared with the above single-omics studies in gilteritinib by Zavorka et al. [88] and Cucchi et al. [153] which focused on a single time point, Joshi et al. [159]. reported the dynamics from early to late resistance, providing new ways of combined therapies to overcome resistance.

Multi-omics-based studies also focused on finding new therapeutic targets for patients with mutant *FLT3-ITD*. Downstream targets of *FLT3-ITD* include Pim kinases, which were associated with resistance to FLT3 inhibitors. A combination of a Pim inhibitor and an FLT3 inhibitor had synergistic cytotoxicity in AML cells with *FLT3-ITD* mutation [161, 162]. Based on this, Hospital et al. [163]. implemented transcriptomic and proteomic methods in profiling Pim2-depleted AML cells with mutant *FLT3-ITD* to study the functions of Pim2 in AML with mutant *FLT3-ITD*. The significantly differentially expressed genes identified were associated with cell death and apoptosis. The proteomics analyses also identified significantly modulated proteins associated with apoptosis and cell death. They then demonstrated that Pim2-dependent apoptosis occurred through expressing Bax and disrupting mitochondria. The transcriptomic and proteomic data also suggested that RSK2 (encoded by the gene *RPS6KA3*) might be a potential target of Pim2 and that knockdown of *RPS6KA3* could reduce the AML cell propagation in mice, indicating RSK2 as a novel therapeutic target for *FLT3-ITD* mutant AML. Later, Kapoor et al. [164]. studied the combined use of clinically active Pim and FLT3 inhibitors both in vitro and in vivo. Results showed that the combination of two inhibitors could abrogate the growth of *FLT3-ITD* AML cell lines, and the combined treatment of Pim inhibitor and FLT3 inhibitor decreased the growth of *FLT3-ITD* mutant cells in mouse xenografts and prolonged animal survival. The effects were not observed in *FLT3-WT* cell lines. The authors believed that clinical tests of combined therapy of Pim and FLT3 inhibitors were worth carrying out.

#### Omics studies associated with venetoclax

**Fig. 5:**
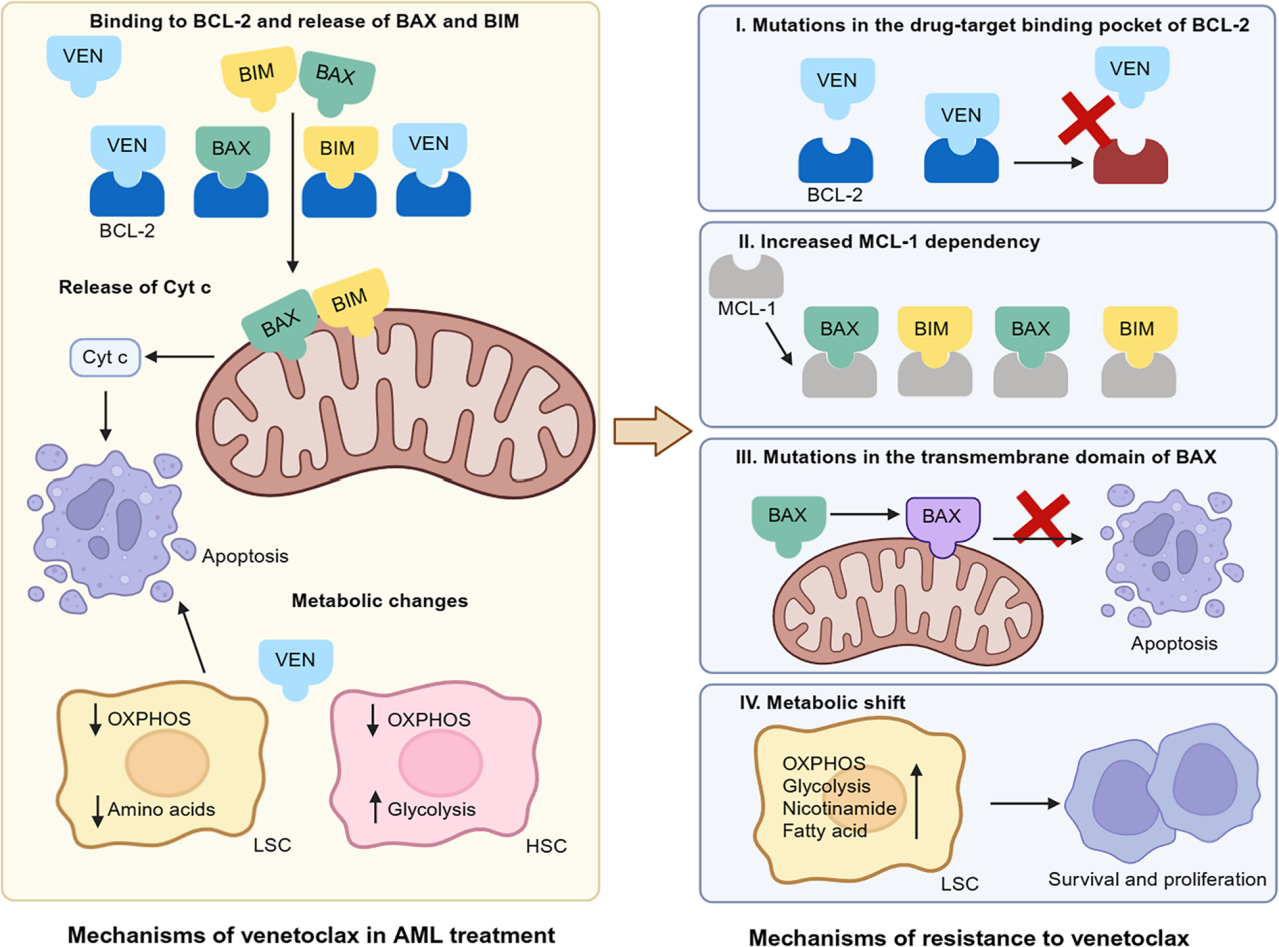
Molecular mechanisms for the resistance of AML to venetoclax. The mechanisms of action of venetoclax are on the left and the mechanisms for the resistance of AML to venetoclax are on the right. Venetoclax binds to BCL-2 and releases BAX and BIM (two pro-apoptotic proteins) from the inhibitory interaction with BCL-2. BAX and BIM increase the permeability of the mitochondrial outer membrane, releasing Cyt c and initiating apoptosis. Venetoclax reduces OXPHOS and amino acid metabolism in LSCs, which also contributes to apoptosis. Mechanisms of resistance include: (I) Mutations of the drug-target binding pocket on BCL-2 prevent the binding of venetoclax to BCL-2. (II) Increased dependency of BAX and BIM on MCL-1 prevents them from locating to mitochondria and therefore decreases the release of Cyt c. (III) Mutations in the transmembrane domain of BAX result in a reduction in BAX-induced apoptosis. (IV) Metabolic shifts include the upregulation of glycolysis, fatty acid oxidation, and OXPHOS to support cell survival

 The therapeutic strategy of venetoclax in combination with chemotherapy has exhibited promising effects in AML patients, although resistance remains a frequent problem (Fig. 5) [25, 165]. Metabolic shift including elevated OXPHOS and glycolysis is an important mechanism in venetoclax-resistance, and metabolomics approaches can assist in deciphering this mechanism and finding possible solutions.

##### Transcriptomics

The resistance to BCL2 inhibitors was associated with MCL1 and BFL1 (two other BCL-2 family proteins) and inhibitors of MCL1 are also under investigation (Fig. 5) [166, 167]. A study aimed at demonstrating the transcriptional signatures of genes from the BCL-2 family (BCL-2, MCL1, and MFL1) was conducted by Lee et al. [168]. Based on RNA-seq datasets from TCGA, BeatAML, and leuceGene, they obtained three signatures including BCL-2, MCL1/BCL-2, BFL1/MCL1 and applied a gene-set selection method in choosing related genes. Through unsupervised clustering, patients were classified into three subtypes. Pathway analyses disclosed that each group had unique enrichment patterns of some major cancer pathways like MAPK or mTORC1 pathway. Therefore, apoptosis-modulating drugs may be selected and combined based on the subtype that the patient is classified into. They also constructed a response prediction system based on the above signatures, which could reliably predict response to venetoclax (AUROC = 0.874), and it was later verified in an independent AML cohort. Their study based on RNA-seq data from a public database successfully classified patients into three subtypes based on the BCL-2 family signature. This is of clinical significance in guiding drug selection and combination therapies and serving as a predictive biomarker for response to venetoclax. They focused only on apoptosis-related signatures and constructed a prediction model, while other mechanisms should be studied to overcome resistance to venetoclax.

Venetoclax displayed significantly improved efficacy in AML patients when combined with other therapies like demethylation therapy azacitidine and MDM2 inhibitor idasanutlin [169, 170]. Lehmann et al. [171]. performed an RNA-seq-based study to monitor the effect of the combination of idasanutlin and venetoclax on AML cell lines and mouse models. The result demonstrated that compared with the single-agent treatments, the drug combination of idasanutlin and venetoclax had superior efficacy. The associated molecular mechanism was re-establishment of p53 tumor-suppressor activity by idasanutlin and induction of mitochondrial apoptosis by venetoclax. The GEP of patients in response to idasanutlin alone displayed huge changes in the p53 pathway and cell cycle arrest (like CCND1 pathway) while that of patients after applying venetoclax or drug combination only showed few genetic changes, which indicated that they functioned mostly at the post-transcriptional level. This study also supported further investigation and trials on combination of venetoclax and idasanutlin.

##### Metabolomics

Apart from glycolysis dependency, cancer cells also rely on OXPHOS for proliferation [172, 173]. Jones et al. [174]. examined the general metabolome profile of LSCs and blasts in 15 primary specimens using MS. Several metabolites related to amino acid metabolism (16 amino acids, 2 intermediates of tricarboxylic acid cycle, and 5 glutathione homeostasis metabolites) were found significantly upregulated in LSCs compared with AML blasts. Consistently, pathway analysis manifested that the amino acid metabolism pathway was significantly enriched in LSCs in comparison to AML blasts. In vitro studies of cell viability and colony-forming ability revealed that depletion of amino acids could reduce the viability of LSCs but had no effect on blast cells. LSCs also exhibited a preferential reliance on amino acids for OXPHOS, being less flexible than AML blasts metabolically. Inhibition of amino acid metabolism with venetoclax and azacitidine reduced OXPHOS and induced cell death of LSCs. However, amino acid metabolism in blast cells was not influenced, suggesting that this treatment selectively affected amino acid metabolism in LSCs. Moreover, LSCs from relapsed AML patients did not display amino acid reliance and were more metabolically adaptive through upregulating fatty acid metabolism, indicating a need for extra therapeutic strategies targeting different metabolic pathways.

The combination of venetoclax and azacitidine has become an alternative option for AML patients, especially for those unfit for chemotherapy. However, there is no reliable marker for predicting the response to venetoclax/azacitidine treatment. Jones et al. [175] later conducted another study that profiled the amino acid metabolome in LSCs of patients resistant to the treatment of venetoclax/azacitidine. Results showed elevation in nicotinamide metabolism in R/R LSCs and OXPHOS was then strengthened through activation of both amino acid metabolism and fatty acid oxidation, which explained the escape of LSCs to venetoclax/azacitidine treatment. They then inhibited nicotinamide phosphoribosyl transferase (NAMPT) and LSCs were eliminated while normal hematopoietic stem cells were not affected. Therefore, targeting nicotinamide metabolism may reduce the OXPHOS of LSCs and help overcome the resistance to venetoclax.

##### Multi-omics

Waclawiczek et al. [176]. integrated clinical information with transcriptomic, proteomic, and functional data to identify biomarkers predictive of venetoclax/azacitidine response. Rather than monocytic-like AML cells (which were resistant to this treatment), LSCs were identified as the primary targets of this treatment and the elimination of LSCs could reflect the treatment outcome. Moreover, LSCs of the refractory group showed perturbed apoptotic dependencies. Based on this, they proposed and further verified a flow cytometry-based scoring system named “mediators of apoptosis combinatorial score” (MAC-Score). The scoring system was composed of the abundance of BCL-2, BCL-xL, and MCL1 in LSCs. Both in the patient cohort and ex vivo experiment, MAC-Score could better predict the 5-AZA/VEN response to venetoclax/azacitidine treatment than the individual BCL-2 inhibitor. This scoring system had a positive predictive value of over 97% in predicting event-free survival (EFS). Even patients with complex karyotypes are fit for this score, showing the high accuracy of multi-omics-based markers.

Jayavelu et al. [177]. performed a comprehensive proteogenomic analysis on BM samples from 252 AML patients at diagnosis. An integrated multi-omics factor analysis (MOFA), which can integrate driver variations in several molecular layers, was performed to demonstrate the proteogenomic landscape of AML. A total of 28 latent factors were identified as driving variations between patients, among which 11 latent factors were associated with variance in several layers, 12 latent factors were only active in the proteome and 5 only active in the transcriptome. They also identified a proteomic subtype with elevated mitochondrial protein expression (Mito-AML) to be significantly correlated with shorter overall survival although patients from this group had favorable risk according to ELN. However, Mito-AML responded better to venetoclax and complex I inhibitors mubritinib and rotenone. In line with Jayavelu’s conclusions, a study by Caplan et al. [178]. performed MS in parallel with RNA-seq analyses in AML mouse models. Thirty-four proteins including several mitochondrial and spliceosome proteins were upregulated but their mRNA levels were unaltered. Overexpression of electron transfer proteins ETFA and ETFB led to dysregulation of mitochondrial processes, and silencing of these two proteins could increase apoptosis, differentiation, and sensitivity to venetoclax.

We have discussed above that MCL-1 overexpression is associated with resistance to BCL-2 inhibitors [179]. Zhang et al. [180]. performed genomic, transcriptomics, proteomic, metabolomic, and methylation analyses on AML cell lines resistant or sensitive to venetoclax. In venetoclax-resistant cell lines, DNA methylation was globally altered and administering of azacitidine could partially overcome the resistance. More importantly, by integrating gene and protein expression data, they observed the upregulation of MCL-1 protein by activating the RAS/MAPK pathway, which was an acquired mechanism of resistance to venetoclax. Results of scDNA sequencing showed clonal selection of RAS-mutated clones in patients treated with venetoclax. Further profiling of metabolites in the venetoclax-resistant cells revealed that mitochondrial respiration was maintained by MCL-1, which supported the survival of leukemic cells. Similar to previous studies [181], they found that pharmacological inhibition of MCL-1 could restore the sensitivity to venetoclax. Their results demonstrated a crosstalk between RAS/MAPK/MCL-1 and venetoclax resistance. The mechanisms of resistance include *NRAS/KRAS* mutations or epigenetic activation of MAPK. The activated MAPK signaling leads to the upregulation and stabilization of MCL-1, which further maintains mitochondrial respiration, promoting AML cell growth.

As a chemo-free and effective therapeutic strategy with a bright future, the venetoclax/azacitidine treatment is under a number of clinical trials, and the associated studies concerning mechanisms and response prediction are also keeping pace with clinical studies. Among a great deal of studies, the multi-omics studies provided irreplaceable information and can guide further deeper studies. Although further verifications are needed, the above multi-omics studies identified potential AML therapeutic targets.

## Omics studies in other approved targeted therapies

### Menin inhibitor

As a newly approved drug, although monotherapy of menin inhibitors showed promising efficacy in clinical practice, many patients failed to respond and relapse was frequent [182, 183]. Therefore, to explore solutions to overcome the refractory to menin inhibitor, Fiskus et al. [184]. performed RNA-seq, scRNA-seq, and assay for transposase-accessible chromatin with high-throughput sequencing (ATAC-seq) in analyzing AML cell lines harboring MLL rearrangement treated with the menin inhibitor SNDX-5613. After treatment, the mRNA expression levels of *ITGAM* and *LYZ* were upregulated while the levels of *HOXA9*, *MEIS1*, *PBX3*, *JMJD1C*, *SENP6*, and *BMI* were downregulated. The same analyses were performed on ATAC-seq data before and after SNDX-5613 treatment and a decrease in *MEF2C*, *MEIS1*, *JMJD1C*, *PBX3*, *SENP6*, *LAMP5*, and *CDK6* was observed. The scRNA profile of AML cells discovered a reduction in cells harboring stem/progenitor signature after treatment. Combining the above data, they found concordant aberrations in mRNA expressions and chromatin accessibility in response to the menin inhibitor. They screened drugs with synergistic effects based on the above conclusion and identified four drugs, inhibitors for BET, MOZ, LSD1, and CBP/p300. All four inhibitors were found effective in an in vitro co-treatment with SNDX-5613. This study based on RNA-seq, scRNA-seq, and ATAC-seq put forward a possible solution to overcome the resistance to the menin inhibitor. As an emerging therapy with great potential, the combination of menin inhibitors with other therapeutic strategies will be investigated and trialed. The four inhibitors selected by Fiskus et al. [184] also need further verification.

### Valproic acid

Hernandez-Valladares et al. [185]. applied a proteomic/phosphoproteomic strategy in identifying proteins associated with response to an AML therapeutic strategy based on all-trans retinoic acid, valproic acid (an HDAC inhibitor), and low-dose cytotoxic therapy. A total of 28 nontreated AML samples were analyzed, among which 11 were responders and 17 were non-responders. Non-responders had elevated levels of proteins associated with processes of hematopoietic and cell death, whereas responders had overexpression in proteins linked to myeloid cell activation, neutrophil degranulation, and M phase regulation. Notably, a quite low overlap between mRNA and protein levels was found in DEPs between the two groups. Phosphoproteomics analysis revealed that responders had increased phosphorylation of proteins like SPTAN1 and ACIN1, apoptosis-related proteins, and substrates of LIMKs and CDKs. To study the early effects exerted by this treatment, they compared phosphorylation proteins of samples before and after the three-day treatment and observed that differentially regulated phosphorylation sites were related to transcriptional and translational regulation and RNA metabolism. Therefore, early prediction of sensitivity to this therapeutic strategy may be conducted by proteomics and phosphoproteomics. Although they did not construct a model or identify a biomarker to predict therapeutic responses, the data for proteome and phosphoproteome in their study are of great value for further investigation on this therapeutic strategy.

### Metabolism associated therapies

The extremely flexible and diverse metabolism leads to the disease aggressiveness as well as the tendency to drug resistance in AML [132]. Targeting metabolic disorders and monitoring clinical responses may be utilized in personalized medicine [133]. Previous studies have demonstrated that AML cells, like other cancer cells, are capable of undergoing compensatory metabolic adaptations in response to the chemotherapies or drugs targeting certain pathways, adding difficulties to sustainable treatment strategies [134, 135].

**Fig. 6:**
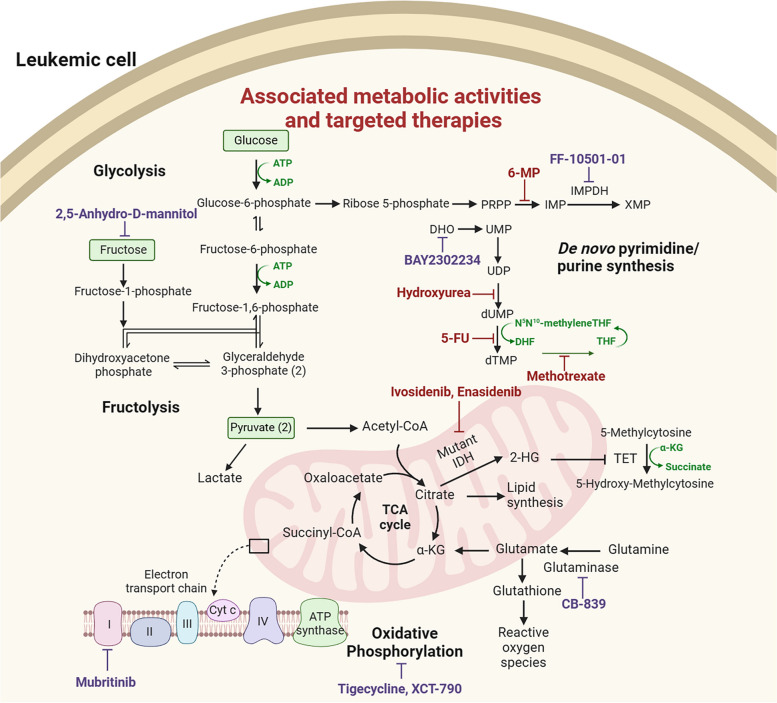
Therapeutic targets and drugs or compounds associated with metabolic pathways in AML. Drugs in red have been approved by the FDA for the treatment of AML. Compounds or drugs currently under investigation or clinical trials are presented in black. ↑ and ⊺ represent activation or inhibition, respectively. Traditional drugs like 6-MP, methotrexate, and hydroxyurea target purine synthesis and suppress cell growth. Mutant IDH1 and IDH2 result in the conversion of α-KG to 2-HG, which promotes epigenetic dysregulation and cellular differentiation blockage. IDH inhibitors, including ivosidenib and enasidenib, can decrease the 2-HG level. Mubritinib targeting complex I and tigecycline and XCT-790 targeting the OXPHOS are under clinical trials. Glycolysis is also an important process in leukemic cell growth although fewer drugs associated with glycolysis have been developed for the treatment of AML

 Drugs targeting the nucleotide biosynthesis are frequently used in AML treatment, as the production of nucleic acid is an important procedure in cell proliferation [186]. Some commonly used drugs (including hydroxyurea, mercaptopurine (6-MP), fluorouracil (5-FU), and methotrexate) inhibit novo purine and pyrimidine synthesis in different nodes of the pathway (Fig. 6) [187–189]. Other drugs targeting the nucleotide biosynthesis under clinical trials included an IMPDH inhibitor FF-10501-01 and an enzyme dihydroorotate dehydrogenase inhibitor BAY24022234, which exhibited promising efficacy [190–192]. Several drugs targeting other pathways have also been proven effective in treating AML patients, including hexokinase inhibitor 2-deoxyglucose and HMG-CoA inhibitor statins [46, 193, 194].

Isocitrate dehydrogenase (IDH) is an enzyme catalyzing the conversion of isocitrate to α-KG and the mutation rate of *IDH1/2* in AML is 5–30% [48] (Fig. 6). The *IDH1/2* mutation is an example of how the dysregulation of metabolic pathways contributing to leukemogenesis became a therapeutic target, which was proven effective in clinical applications [195]. DiNardo et al. [196]. measured the 2-HG concentration in serum samples derived from 223 AML patients through reverse-phase LC-MS and discovered that 2-HG levels were increased in patients with *IDH1/2* mutation and being a potential diagnostic marker for *IDH1/2* mutation, presenting a specificity of 90.7% and a sensitivity of 86.9%. As a prognostic marker, patients harboring *IDH* mutation with 2-HG levels > 200 ng/mL at CR had a significantly shorter OS compared to those with 2-HG ≤ 200 ng/mL. In the context of treatment, some drugs targeting this mechanism have been approved by the FDA and some were in clinical trials. Glutaminase inhibitor CB-839 was effective in reducing 2-HG concentration and suppressing the growth of AML cells [197]. However, 2-HG levels were not detectable in some patients treated with IDH inhibitors, even in those with significant responses [20], suggesting the presence of other mechanisms in the association between mutant *IDH* and leukemogenesis. Although both mutant *IDH1* and *IDH2* promote the accumulation of 2-HG, differences occur between the metabolism of mutant *IDH1* and mutant *IDH2* [198, 199]. Bassal et al. [200]. found that only mutant *IDH1* could cause the mutual exclusivity between electron transport chain complex I variants.

In a phase I/II clinical trial of CB-839 combined with azacitidine in treating advanced MDS (NCT03047993), promising efficacy and safety were demonstrated [201]. However, in a phase I study of administering CB-839 on relapsed/refractory leukemia patients (NCT02071927), only few patients had a significant decrease in blast counts [202]. Compared with the glutaminase inhibitor, the IDH inhibitor had better treatment efficacy in clinical trials. Enasidenib inhibits neomorphic IDH2 and thus reduces 2-HG level. This drug was approved by the FDA for the treatment of R/R AML in 2017 [20, 203, 204]. Meanwhile, the IDH1 inhibitor ivosidenib was also approved for treating R/R AML in 2018 [21]. Both IDH1 and IDH2 inhibitors had superior clinical outcomes compared to previous outcomes of patients with R/R AML [205, 206], demonstrating a successful example of translating studies of dysregulated metabolism to effective targeted therapy.

Although a limited number of metabolomics-based studies have been conducted in AML, several drugs with great potential have been applied to clinical use. We believe that metabolomics has a promising future in developing therapeutics for AML.

### Omics in immunotherapy and cellular immunotherapy

Immunotherapies including chimeric antigen receptor (CAR) T cell therapy, antibody-drug conjugates (ADCs), programmed cell death-1 (PD-1), and programmed cell death ligand-1 (PD-L1) have been applied in some AML patients. Although not as commonly used as chemotherapy or targeted therapy, immunotherapies still showed efficacy in many cases [207]. A few omics-based studies were conducted in immunotherapy.

#### Omics in CAR-T therapy

Approved by FDA in 2017, CAR-T therapy was the breakthrough in cancer therapy, both in solid tumors and hematological cancer. Unfortunately, due to the lack of appropriate target, CAR-T therapy has not been widely applied in AML patients. Some CAR-T cell target antigens may also inhibit normal hematopoiesis, like CD123, and are unsuitable for clinical use [208]. RNA-seq helps to evidence the subpopulation of post-chemotherapy tumor cells and identify the surface antigens that are expressed in malignant cells but lacking in healthy cells.

Calvino et al. [209] performed RNA-seq to compare the phenotypic and functional differences between CD4^+^ and CD8^+^ CD33-CAR-T cells and CAR-T cells from AML patients and healthy controls, the results were similar between the two groups, especially in senior CAR-T cells. Gottschlich et al. [210] obtained RNA-seq data of 500,000 single cells from 15 AML patients and nine healthy controls to predict the target antigens and successfully identified two target antigens: colony-stimulating factor 1 receptor (CSF1R) and cluster of differentiation 86 (CD86). The follow-up in vitro and in vivo validation confirmed that CSF1R and CD86 expressed broadly on AML blasts responded well in CAR-T cells and had minimal toxicities to healthy cells as well.

#### Omics in ADCs

Antibody-drug conjugates are the delivery of a potent toxin to the targeted cells by the specificity of antibody. The ADC targeting CD33 gemtuzumab ozogamicin, which was approved by the FDA in 2000, was ultimately eliminated for toxicity and low benefit [211]. However, gemtuzumab ozogamicin was reapproved recently by implementing a fractionated dosing regimen in the clinic [212]. Jiang et al. [213] analyzed an ADC target, C-type lectin domain family 12 member A (*CLL1* or also known as *CLEC12A*) that was expressed on LSC and AML blasts but not on normal HSCs. They performed RNA-seq to analyze the transcript levels of *CLL1* from LSCs and AML blast cells and normal HSCs. *CLL1* is expressed highly in AML cells. For healthy controls, *CLL1* is expressed normally in their bone marrow tissue while low in other normal healthy tissues. The compelling expression profiles in AML blasts made *CLL1* an ideal ADC therapeutic target. Jiang et al. then developed CLT030 (CLL1-ADC) which was stable in the bloodstream and could release DNA-binding payload only after ADC binds to CLL1-expressing tumor cells.

#### Omics in PD-1/PD-L1

PD-1/PD-L1 signaling was involved in leukemia development [214] and anti-PD-1/PD-L1 treatments were effective in some cases [215]. Abbas et al. [216]. used single-cell functional proteomics profiling in identifying predictors for treatment responses to anti-PD-1 therapy in AML patients. The results uncovered that the main drivers of the enhanced polyfunctionality index of the pretherapy CD4^+^ subset were tumor necrosis factor-α (TNF-α) and interferon-γ (IFN-γ).

### New targets in AML identified by omics studies

#### Transcriptomics

As Li et al. [217]. reported in their study, expression of dihydropyrimidinase-like 2 (*DPYSL2*) was downregulated in AML cells resistant to homoharringtonine and analyses of TCGA database uncovered that high expression of *DPYSL2* was correlated with JAK/STAT pathway and was associated with worse OS of AML patients [218]. They also performed RNA sequencing for AML cell lines with the mock or *DPYSL2* knockdown and the gene expression was distinct between these two groups. The PI3K‐AKT pathway was significantly downregulated in the *DPYSL2* knockdown cells. Therefore, they identified *DPYSL2* as an oncogene functioning by regulating the JAK2/STAT axis and as a potential target in treating AML.

As an AML entity defined by fusion genes, AML with MLL rearrangement is a research hotspot in targeted therapy and DOT1L and menin are the most representative targets. It was found that DOT1L was involved in the development of MLL rearrangement AML and promoted the gene expression of MLL as well as its fusion partners AF4, AF9, AF10, and ENL [219, 220]. EPZ-5676 is currently a clinically used DOT1L inhibitor, which selectively inhibits cell proliferation, and promotes cell apoptosis and differentiation [221]. Long-term use of EPZ-5676 leads to drug resistance and this drug is generally used in synergies with other anti-AML drugs [114, 222]. Klaus et al. [223]. used EPZ-5676 in the combination with arabinofuranosyl cytidine (Ara-C) or daunorubicin to induce synergistic and durable anti-AML proliferative effects. Yi et al. [221]. reviewed the therapeutic strategies against DOT1L in treating AML with MLL rearrangement. Combination of the DOT1L inhibitor with other epigenetic therapies such as menin inhibitors, SIRT1 activators, and BRD4 inhibitors had enhanced effects, and more drugs targeting DOT1L will be moved into clinical trials in the future [224–226].

STAG2, SMC1A, SMC3, and RAD21 consist of the cohesin complex and about 50% of patients harboring *NPM1* mutation also have a mutation in the cohesin complex [227, 228]. Meyer et al. [229]. constructed murine models harboring *NPM1* and *SMC3* mutations and performed RNA sequencing. Results showed that *SMC3* mRNA expression level was not altered by *NPM1* and the combined *NPM1* and *SMC3* mutation uniquely altered the whole transcriptome with increased expression of genes associated with actin cytoskeletal regulation that was not found with either single mutation. Further analyses based on transcriptomic data revealed an upregulation of *DOCK1* (a Rac1/2 nucleotide exchange factor) and *ELMO1* (two actin regulators) in samples harboring both mutations. Among the two upregulated genes, *DOCK1* was specifically upregulated in hematopoietic stem/progenitor cells (HSPCs) with *NPM1* (+) and *SMC3* (+). Knocking down *DOCK1* could significantly reduce the growth of AML cells with both mutations, indicating it is a specific target for *NPM1*(+) AML. Meanwhile, increased Rac activity was detected in cells harboring both mutations compared with *NPM1* mutation only. Pharmacological inhibition of Dock1 by CPYPP (a commercially available small-molecular Dock inhibitor) and inhibition of Rac by EHT 1864 (a pan-Rac inhibitor) could both enhance apoptosis of leukemic cells. Instead of considering *NPM1* only, their RNA-seq-based study focused on both *NPM1* and mutations in the cohesin complex and identified Dock1/Rac as a druggable target for these AML patients.

Hu et al. [230]. obtained datasets of RNA expression of 510 samples (173 AML patients and 337 healthy donors) in TCGA-GTEx. A total of 4164 genes were significantly upregulated and 7756 were significantly downregulated. Further statistical analyses identified 10 genes significantly associated with OS and among them, *LYPD3* (Ly6/PLAUR domain-containing protein 3) independently predicted poor prognosis in AML. LYPD3 is one of the high-glycosylated cell surface proteins that are associated with the carcinogenesis of several solid tumors [231–234]. Knockdown of *LYPD3* could suppress cell proliferation and induce apoptosis. Gene set enrichment analysis results indicated that the p53 signaling pathway, PI3K-AKT signaling pathway, and E2F signaling pathway were enriched for high *LYPD3* expression, and the participation of LYPD3 in AML progression was confirmed to be involved in the p53 and PI3K-AKT signaling pathways. Therefore, they identified *LYPD3* as an oncogene of AML with therapeutic potential.

Dysregulated mitochondrial oxidative phosphorylation (mtOXPHOS) is associated with leukemogenesis and is also important in promoting disease progression and drug resistance [235, 236]. Estrogen-related receptor α (ERRα) is an orphan nuclear receptor associated with mitochondrial energy production [237]. To investigate the regulator of mtOXPHOS in AML and explore its associated treatments, Seo et al. [238]. performed sc-RNA sequencing on primary AML cells from patients as well as in vitro and ex vivo analyses in studying the role of *ERRα* in regulating mtOXPHOS in AML. Cells expressing *ERRα* were significantly enriched for mtOXPHOS in comparison to those without *ERRα* expression, and mtOXPHOS-related genes also had higher expression levels in cells with *ERRα* expression than in normal cells or other leukemic cells. Pharmacological inhibition of ERRα with XCT-790 could promote anti-leukemic effects through mtOXPHOS suppression. The mtOXPHOS pathway and mitochondrial genes were also downregulated after XCT-790 treatment. Their study identified ERRα as a potential therapeutic target through blocking mtOXPHOS in AML. As an important metabolic process, OXPHOS will be discussed later in the metabolomics section. However, this study discovered a metabolism-related drug target based on scRNA-seq approaches, indicating the close connection between different omics studies and the rationality of adopting different omics approaches in AML studies.

Many novel targets have been identified by transcriptomics. However, some studies only analyzed GEP and GEP-associated pathways, and others conducted in vitro or ex vivo validations. Some targets may only be associated with mechanisms of leukemogenesis or relapse and have limited therapeutic value. Even though transcriptomics adds to our understanding of the expression and functions of genes, there is still a long way to go from the potential targets identified by transcriptomic studies to result in clinical treatment.

#### Proteomics

Profiling of proteome to detect DEPs can provide clues for drug targets. Analyses of PTMs in proteins contribute to exploring alterations in downstream signaling pathways that are driven by genomic changes. The technology of MS-based proteomics has enabled comprehensive profiling of proteins and deep dissection of signaling pathways in AML. Benefiting from this technology, some new drug targets in AML have been identified.

In a study conducted by Casado et al. in 2013 [239], the phosphoproteome in 20 primary AML samples was profiled. They implemented the Integrative Inferred Kinase Activity algorithm (INKA) in identifying hyperphosphorylated kinases with high activities as treatment candidates for kinase inhibitors [240]. Several kinase subgroups (including PI3K, CDKs, and ERK) were selected to predict the sensitivity to P13K and mTOR inhibitors. They later conducted another study combining phosphoproteomic data with mutational and immunophenotypic data in identifying determinants for primary AML’s response to PKC/FLT3, MEK, PAK, CK2, and MAPK inhibitors [154]. Protein phosphorylation was found to have a positive correlation with the expression of differentiation-related makers. Highly differentiated samples had higher expressions of kinases and signal transduction regulators. Therefore, more differentiated cases presented increased activities of kinases and their downstream factors. These cases were more sensitive to the above inhibitors, establishing a link between kinase activities, cell differentiation, and response to kinase inhibitors. Compared with a single mutation or a single protein, pathways can better reflect the general status of a disease or the response to certain treatments. Although the effects of the inhibitors they selected need further verification, their studies demonstrated a possibility of using LC-MS/MS in predicting drug response by measuring the activities of targeted pathways.

Buet et al. [241]. profiled the tyrosine-phosphorylated proteins in *KIT*-mutant murine leukemia proerythroblasts. They first identified Shp2 and Stat5 as proximal effectors of KIT through LC-MS/MS and then validated in leukemic cells that Shp2 and Stat5 were persistently phosphorylated depending on mutant *KIT* activities. To further provide a molecular rationale for drug targets, they found that depletion of *Shp2* or *Stat5* and inhibition of PI3 kinase or MEK/ERK activities at the same time could suppress leukemic growth. Therefore, the combination of the NVP-BEZ235 (PI3K inhibitor) and obatoclax (a BCL-2 inhibitor) was proven to synergistically inhibit leukemia cell growth, providing evidence for new treatment options targeting dominant signaling pathways. Van Alphen et al. [242]. performed phosphotyrosine enrichment-based label-free quantitative phosphoproteomics on 16 AML cell lines and INKA algorithm was used to identify phosphorylated candidate kinases for kinase inhibitors. Apart from driver kinases related to their activating mutations that were already present in cell lines, they also pinpointed several RTKs drivers undetected by standard molecular analyses through INKA. These cell lines were highly sensitive to specific kinase inhibitors selected based on the above INKA analyses. Furthermore, the hyperactivation of FLT3 in the MM6 cell line detected by INKA was verified in two clinical AML samples. This approach identified hyperactive kinases as potential drug targets based on phosphoproteomics and INKA analysis, demonstrating the possibility of selecting specific kinase inhibitors for individual AML patients.

Proteomics approaches also contribute to finding appropriate drug combinations in treating AML patients. To find a novel treatment option that might compensate for the short duration of using FLT3 inhibitors alone, Murray et al. [243]. examined the proteome and phosphoproteome profile of AML blasts deriving from 7 patients (4 with mutant *FLT3* and 3 with *WT FLT3*). In *FLT3* mutant samples, proteins with increased phosphorylation included DNA-PKcs (PRKDC), X-ray repair cross-complementing 5 (XRCC5), XRCC4, and tumor protein p53 binding protein 1 (53BP1), which were associated with the error-prone DNA-PK-dependent nonhomologous end-joining pathway. Mutant FLT3 samples exhibited increased autophosphorylation of DNA protein kinase (PRKDC) at S261, which was sensitive to FLT3 inhibitors. Both FLT3 inhibitors midostaurin and sorafenib, when combined with DNA-PK inhibitor M3814, exhibited a synergistic effect in reducing the growth of mutant FLT3 cell lines but not in WT FLT3 cell lines. Further tests in vivo preclinical AML murine model confirmed the combined effects of sorafenib and M3814. Mice treated with combined therapy survived longer than those treated with sorafenib alone or M3814 alone. Their study based on phosphoproteomic data rationalized the combined use of FLT3 and DNA-PK inhibitors in *FLT3* mutant AML cases.

Another study by Koschade et al. [156]. identified and rationalized the combined use of autophagy inhibitors and FLT3 inhibitors based on functional translatome proteomics and phosphoproteomics. Phosphoproteome profiling unveiled elevated phosphorylation of proteins associated with mTOR signaling and autophagy after treatment with quizartinib, crenolanib, or gilteritinib. Further studies in AML cell lines showed that drug-induced autophagy by FLT3 inhibitors was observed in those with mutant *FLT3*, but not those with *WT FLT3*, which induced autophagy through an AKT-mTORC1-ULK1 pathway and involved the decreased phosphorylation of mTOR and ULK1. The sensitivity of the *FLT3-ITD* mutant cells to FLT3 inhibitors was increased after inhibition of the drug-induced autophagy through chemical and genetic approaches. Consistently, simultaneously treating blast cells from *FLT3* mutant AML patients with FLT3 inhibitors and autophagy inhibitors could synergistically reduce the viability and proliferation of cells. Their research demonstrated the possibility of using functional translatome proteomics and phosphoproteomics in investigating combined therapies for AML which may overcome drug resistance. Several other studies also reported the dysregulation of autophagy in AML treated with FLT3 inhibitors and the potential of overcoming resistance through interfering autophagy [244–246]. Autophagy is dysregulated in AML and is a hotspot in AML research with a number of emerging drug targets [247]. Autophagy is also closely related to metabolic abnormalities and further implementing metabolomics approaches may extend the conclusion of Koschade et al. [156]. and provide more evidence for the combination use of FLT3 and autophagy inhibitors.

Allert et al. [248]. applied multilayered proteome analyses in AML cell lines to study the acquisition of resistance to midostaurin. In the early stage of resistance, 150 proteins were downregulated and 104 were upregulated. Among the upregulated ones, LPXN (leupaxin) was found to be induced in both early and late resistance. LPXN is a transcriptional coactivator that regulates cell migration and adhesion and is induced together with PTK2B (a tyrosine kinase phosphorylating LPX) in the early stage of resistance. Analyses using nascent proteomics showed that pharmacological inhibition of PTK2B could revert the midostaurin resistance-associated alterations. PTK2B inhibition could also decrease the cell migration and adhesion of midostaurin-resistant cells. Combined treatment using gileritinib and PTK2B inhibitor defactinib demonstrated better effects than using either drug alone in xenograft mouse models. Their study innovatively demonstrated the dynamics of proteome after midostaurin treatment and found a synergistic therapy that might overcome resistance to FLT3 inhibitors at an early stage.

Selinexor is the inhibitor of the nuclear export protein exportin-1 (XPO1) and can restore p53 accumulation, which has been demonstrated to provide promising effects in AML treatment [249]. To identify rational drug combinations for selinexor, Emdal et al. [250]. used phosphoproteomics in profiling signaling responses to selinexor in 20 primary AML patient samples after 6 h treatment with selinexor. Among the 20 samples, 9 were responders of selinexor and 11 were non-responders. It is worth mentioning that genomic aberrations like *FLT3* and *WT1* were poorly correlated with selinexor response. In responders, selinexor significantly upregulated the phosphorylation of *TP53* at S315, which is involved in transcriptional activation of *TP53* [251]. However, in those classified as non-responders of selinexor, the drug significantly upregulated the phosphorylation of FOXO3A at S253, which is an AKT site retaining cytoplasmic sequestration of FOXO3 and inhibits its proapoptotic activity [252]. Therefore, enhancing *TP53* might potentiate the effects of selinexor and inhibiting the phosphorylation of FOXO3A may reduce resistance to selinexor. Nutlin-3a is an MDM2 inhibitor that prevents p53 ubiquitination and proteasomal degradation, elevating its protein level and enhancing its tumor-suppressor function. Treating AML cell lines without *TP53* mutation and sensitive to selinexor with nutlin-3a, a synergistic effect was produced when nutlin-3a stabilized p53, thus enhancing the selinexor-induced cell death. Additionally, in selinexor-resistant cell lines, the combination of MK-2206 (an AKT inhibitor) with selinexor overcame the resistance of selinexor through an increase of nuclear localization of FOXO3A, which was not observed in MK-2206 treatment alone. Their study identified potential combinations of drugs with selinexor to overcome its resistance and enhance the treatment effects based on phosphoproteome profiling.

For purpose of overcoming resistance to clinically used therapies or searching for novel therapeutic options, several drug targets have been identified based on proteomics. Some were discovered only based on high-throughput sequencing and pathway enrichment analyses and others were further validated with in vitro or ex vivo experiments. More studies are needed before these potential novel drugs can be applied to clinical use.

#### Metabolomics

A recent study by Thomas et al. [253]. reported that acetyl CoA carboxylase 1 (ACC1) was a synthetic lethal metabolic target for mutant *IDH1* while mutant *IDH2* did not yield this effect. Because ACC1 is a lipid synthesis enzyme, they performed LC-MS-based metabolomic analyses on primary AML blasts with or without mutant *IDH1* and focused on lipid metabolism. They observed a reduction in fatty acids and the switch to β-oxidation in cells with *IDH1* mutation, indicating the fatty acid reliance of AML cell metabolism caused by mutant *IDH1*. The reliance on fatty acids was further verified through tests of a lipid-free diet in mouse models, which suppressed the growth of AML cells with mutant *IDH1* while cells with mutant *IDH2* were not affected. Pharmacological inhibition of ACC1 improved the effects of both ivosidenib and venetoclax in AML cells with mutant *IDH1*. Therefore, for patients with *IDH1* mutation, targeting ACC1 in combination with ivosidenib or venetoclax may be a potential therapeutic strategy.

#### Multi-omics

We have summarized studies implementing single omics approaches in refining the molecular subgroup of AML. We believed that integrating data from different omics layers would provide better classifications associated with prognosis prediction and drug target identification.

Dysregulated protein function resulting from mislocalization of proteins to the nucleus may disrupt their functions and promote cancer development by affecting normal hemopoietic processes or diminishing the functions of tumor suppressors [254]. To study the influence of aberrant protein localization on AML, Alanazi et al. [255]. examined the protein abundance in nuclei of AML blasts derived from 15 newly diagnosed AML patients using LC-MS/MS and performed parallel transcriptome analyses in correlation with the proteome data. RNA-seq identified a total of 40 transcription factors with differential expression, but they were not correlated with protein levels, which emphasized the necessity for performing proteomics at the subcellular level. They identified 113 proteins with significant changes in abundance in AML blasts, which formed multiple complex interaction networks. The networks were significantly correlated with transcription regulation, mRNA processing, and mRNA stabilization. Among the identified proteins, S100A4 was the highest differentially expressed protein. S100A4 is a calcium-binding protein belonging to the *S100* multigene family, it is reported to be associated with poor prognosis in some solid tumors [256–258]. Overexpression of S100A4 was validated in another AML cohort. *S100A4* knockdown could impair the growth of AML cells through programmed cell death while exerting no effects on normal cells. Their study found abnormal expression of transcription factors that were unable to detect at the mRNA level and identified S100A4 as a potential therapeutic target. As we discussed above, both mRNA and the protein level of S100A8 are associated with chemoresistance and resistance to FLT3 inhibitors. Therefore, S100 family may be worthy of further study as drug targets.

For some AML patients harboring cytogenetic and genetic alterations with adverse prognoses (complex karyotypes, KMT2A-rearrangements, monosomy karyotypes, and *TP53* mutations), effective targeted therapies are not available. To find possible treatment options for these patients, Casado et al. [259]. performed proteome and phosphoproteome profiling for 74 AML patients with adverse prognoses and additional transcriptomics analysis for 39 of these cases. A total of 550 drugs were tested for ex vivo responses. Integrating omics data, they divided KMT2A-rearranged AML into two biologically distinct groups MLLGA and MLLGB. Elevated levels of DOT1L phosphorylation and *HOXA* gene expression and increased CDK1 activity were detected in MLLGA cases compared with MLLGB cases and cases without KMT2A rearrangement. The MLLGA cases also demonstrated significantly high sensitivity to 15 compounds including inosine-5-monosphosphate dehydrogenase (IMPDH), mitotic kinaseinhibitors, and several genotoxic drugs. This study reclassified AML cases with KMT2A rearrangements into two biologically distinct groups based on multi-omics data. It also demonstrated the sensitivity of several drugs including IMPDH inhibitors in KMT2A patients with MLLGA signature. Their study based on transcriptomics and proteomics helped to find suitable drugs for AML patients within the adverse risk group. Although not verified in large cohorts, the clues in potential treatment strategies are valuable for these patients with poor survival.

Associated with poor prognosis, the extramedullary infiltration (EMI) is still poorly studied with almost no effective treatments. However, EMI samples are suitable for multi-omics studies. Yang et al. [260]. performed scRNA sequencing on EMI and BM samples from one AML patient harboring pervasive leukemia cutis and found increased macroblasts, promonoblast, and monoblasts, and decreased granulocyte-monocyte progenitor-blasts. The macroblasts expressed high levels of C1Q (including C1QA, C1QB, and C1QC). In an EMI AML patient cohort, they validated that high C1Q expression was present before EMI manifestations. Univariate and multivariate analyses defined C1Q as a marker for poorer prognosis. To demonstrate the expression status of C1Q in different courses of disease, RNA sequencing and quantitative proteomic analyses were performed on samples of healthy donors, early-stage AML patients, and AML patients during treatment (some with relapse). The C1Q level in AML samples was surprisingly lower than those in healthy donors whereas its expression level was high in samples from early relapse patients, indicating that it was upregulated later in disease progression and may be associated with early relapse. Univariate and multivariate analyses demonstrated the adverse prognosis significance of C1Q expression. Further functional analyses showed that C1Q was associated with infiltration and migration of leukemia cells, and may promote chemoresistance. Their study implemented different omics approaches and identified C1Q as a biomarker for poor prognosis and EMI. More importantly, they demonstrated the dynamic expression of C1Q from diagnosis to relapse. The upregulation of C1Q in the progression stage made it a possible druggable target.

Passaro et al. [261]. performed high-throughput omics analyses including transcriptomics and proteomics on primary AML samples, AML mouse models, and cell lines, profiling hematopoietic stem cells as well as the BM microenvironment of hematopoietic stem cells (referred to as the “niche”) [262]. In total, eight functional clusters of transcripts based on GEP were identified in the BM niche cells, with each cluster enriched for different functions. They then engrafted immunodeficient mice with patient-derived AML samples harboring different cytogenetical abnormalities and examined the alterations of BM niche components. Cluster 2 (enriched for endothelial functions) remained stable while several signaling pathways including Notch, NF-κB, and Wnt were significantly altered, as were also shown in other clusters (like 3 or 7) which lost their original expression pattern. They further analyzed the proteome in the BM secreta of AML xenografts and integrated it with the transcriptomic data. Significant alterations in signaling nodes related to multiple stromal types were observed, indicating distinct local regulation. Their study provided a great amount of omics data concerning the BM microenvironment of hematopoietic stem cells and demonstrated the general pathological hallmarks of niche in AML disease. Their data may be a foundation and repository for further identification of biomarkers or drug targets.

Genomic studies have identified *SRSF2*, *SF3B1*, and *U2AF1* as recurrent mutations in splicing factors in around 10% of AML patients [263, 264]. Liu et al. [265]. studied the dysregulation of splicing factor expressions and proteome alterations resulting from alternative splicing in AML LSCs. After analyzing the gene expression data of 203 mRNA splicing factors from GEO, they identified RNA-binding motif protein 17 (*RBM17*) as the only factor that was related to poor prognosis and enriched in LSCs. Its elevated expression was also validated in TCGA and BeatAML datasets. Knockdown of *RBM17* impaired the cell growth and colony-forming of LSCs and the RBM17-mediated splicing events were associated with leukemia propagation. To determine the existence of protein downregulations caused by RBM17-mediated splicing, they further performed LC-MS-based proteomics in profiling AML cells after *RBM17* knockdown. A total of 1157 proteins exhibited significant changes and were enriched for cell division, RNA processing, DNA replication and repair, autophagy, protein folding, and vesicle organization. Notably, they also identified 13 proteins downregulated upon *RBM17* knockdown. Analyses of multi-omics data unveiled that *RBM17* knockdown resulted in the upregulation of the translation initiation factor EIF4A2 (eukaryotic translation initiation factor 4A2), which was highly expressed in LSCs. They examined the proteome profile of EIF4A2-depleted AML cells and observed effects similar to *RBM17* knockdown, including suppression of downstream proteins related to leukemic cell growth. Therefore, they believed that RBM17 supports the survival of LSCs through enhancing pro-LSC transcripts like EIF4A2, and RBM17 and EIF4A2 were possible choices for targeting LSCs in AML treatment.

*TP53* mutation defines a distinct entity of myeloid malignancies and the presence of mutant *TP53* in MDS or MPN (myeloproliferative neoplasms) indicates a higher chance of leukemic transformation and worse prognosis [263, 266, 267]. *TP53* mutation is involved in clonal evolution and subsequent acquisition of aberrant LSCs [268, 269]. Rodrigeuz-Meira et al. [270]. performed single-cell multi-omics analyses (including NGS, SNP arrays, and scRNA-seq) on HSPCs from both *TP53*-mutant sAML patients and WT *TP53* AML patients. HSPC clones with *TP53* “multihit” were enriched for LSC-associated transcriptions and the clones were observed at leukemic transformation. Meanwhile, WT *TP53* pre-LSCs clones were significantly associated with erythroid-related transcription. They also compared the HSC signature between *TP53*-sAML and *de novo* AML and observed erythroid-biased differentiation in *TP53*-sAML. A 44-gene signature named “p53LSC-signature” was identified and verified to be reliable for survival prediction, including both *TP53*-mutant and WT *TP53* patients. Their study demonstrated that *TP53* mutation drove leukemic transformation through multi-omics profiling of *TP53*-mutant HSPCs. They also constructed a *TP53*-related gene expression signature which could predict prognosis and assist the selection of therapeutic strategies.

As we discussed above, OXPHOS is an important process in leukemogenesis and is also essential for the survival of LSCs [271]. Therefore, targeting OXPHOS may be a possible treatment for AML LSCs. The sirtuin (SIRT) protein family is associated with energy metabolism in cancers and was studied in cancer stem cells in several solid tumors [272–274]. Among them, SIRT3 has the functions including suppressing ROS levels and regulating fatty acid metabolism and glycolysis [275–277]. Based on these, O’Brien et al. [278]. speculated and verified SIRT3 to be an essential target involved in LSC survival and functions. Knockdown and inhibition of SIRT3 impaired the function of LSCs but did not affect normal BM cells. They then performed multi-omics analyses including transcriptomics, proteomics, and lipidomics for AML LSCs derived from patients. Multi-omics data demonstrated that SIRT3 regulated fatty acid oxidation as well as OXPHOS and affected LSC functions. Therefore, they identified SIRT3 as a potential therapeutic target that interferes OXPHOS in AML LSCs and the combination of data from different biological layers made their findings more convincing.

Single-cell multi-omics approaches are developing fast and are capable of examining surface proteins along with other abnormalities, including chromatin accessibilities (ATAC-seq) [279], mutational profiling (single nucleotide variations as well as structural variants) [280] and transcriptomics dysregulations (CITE-seq and epitopes by sequencing) [281, 282]. Metabolic information can also be integrated through measuring mitochondrial marker mutations (genotyping of transcriptomes, TARGET-seq, and MutaSeq) [283–285]. Recently, Beneyto-Calabuig introduced a method named CloneTracer which could add clonal resolution to scRNA-seq data [286]. Therefore, we believe that implementing single-cell multi-omics approaches in studying the component and clonal evolution of HSC and LSC will deepen our understanding of AML origins and provide clues for targeting LSCs of AML in the future.

Successful intervention on AML with targeted therapies guided by using multi-omics analyses remains challenging when faced with this rapidly progressing disease. However, encouraging achievements have been reached based on these approaches in the field of AML. In the future, more studies will dig deeper beyond the genetic aberrations of AML and the multi-omics will play an irreplaceable role.

## Conclusions and perspectives

### Summary

Despite the already comprehensive MICM criteria in AML diagnosis and classification, conducting personalized treatment still requires more biological information. Studies based on genomics, transcriptomics, proteomics, and metabolomics, both individually and integrated, have tremendously deepened our understanding of AML and expanded the horizon from the traditional morphologic and cytogenetic perspective. Comprehensive clustering of the differential molecules in AML based on these studies might complement the current classification of AML by defining new pathological subtypes which further link to prognosis predictions and specific therapeutic vulnerabilities. Subtypes based on gene or protein expression profiles extended the cytogenetic and mutation-defined subtypes, helping to distinguish individual patients with similar mutations or karyotypes. Subtypes based on the prediction of prognosis using biomarkers identified by omics studies may be more valuable in clinical practice in guiding the selection of therapeutic strategies, especially for the intermediate-risk patients (Table 2). Not only the mechanisms under treatment resistance and possible solutions have been studied through multi-omics methods, but new drug targets are also being identified and tested (Table 3). Several targeted drugs have already been used in clinical practice or are under clinical trials. Precision medicine requires individualized therapy and subtype classification as well as drug selection for patients harboring different omics signatures will sure be the future direction of precision medicine. Although current omics-based studies mostly focus on molecular subtypes and druggable targets in signaling pathways, there are still big gaps in omics studies concerning transplantation and cell therapies. We believe that omics approaches will soon cover more areas of AML researches.

**Table 2:** Potential biomarkers in AML identified by omics-based studies

Biomarker	Samples	Clinical significance	Major omics approaches	Literature
LOXL1, FAM81A, mTORC1, KRAS	BM samples from 12 AML patients	predictor of relapse	RNA-seq-based transcriptomics	Zhai et al. [30]
a 6-gene model: NFKB2, NEK9, HOXA7, APRC5L, FAM30A, and LOC105371592	421 AML patients from BeatAML and 136 from TCGA for training, 215 from GEO for validation	predictor of prognosis and relapse	RNA-seq-based transcriptomics	Guo et al. [40]
CBFA2T3::GLIS2	Blasts from 14 AML patients for training, BM samples from 62 patients for validation	predictor of poor prognosis	RNA-seq-based transcriptomics	Gruber et al. [56]
ADAM8	CD34^+^ cells from 8 MDS patients for training, samples from 29 MDS and sAML patients for validation	predictor of progression from MDS to AML	microarray-based transcriptomics	Vasikova et al. [64]
TPOR	CD34^+^ cells from 8 MDS patients for training, samples from 29 MDS and sAML patients for validation	predictor of MDS progression and biomarker of sAML	microarray-based transcriptomics	Vasikova et al. [64]
antioxidant genes	BM samples from 97 MDS patients and 25 healthy controls	predictor of MDS progression and biomarker of sAML	RNA-seq-based transcriptomics	Picou et al. [67]
mTOG	BM samples from 53 MDS and sAML patients and 9 healthy controls	predictor of progression from MDS to AML	MS-based proteomics	Guzzi et al. [70]
PAIP1	BM samples from 53 MDS and sAML patients and 9 healthy controls	predictor of progression from MDS to AML	MS-based proteomics	Guzzi et al. [70]
CXCL4, CXCL7	Serum samples from 138 MDS patients and 112 controls	predictor of progression from MDS to AML	MS-based proteomics	Aivado et al. [71]
FBXO11	Samples from 15 sAML patients	predictor of progression from MDS to AML	LC–MS/MS-based proteomics	Schieber et al. [72]
MOES, EZRI, and AIFM1	Samples from 5 AML patients	biomarker of sAML	MS-based proteomics	Braoudaki et al. [73]
HOXA9	BM samples from 11 AML patients	predictor of poor prognosis	microarray-based transcriptomics	Golub et al. [77]
PEAR1	Samples from 108 AML patients for training, 252 for validation	predictor of poor prognosis	RNA-seq-based transcriptomics	Bottomly et al. [81]
FLI1	Samples from 511 AML patients	predictor of relapse	MS-based proteomics	Kornblau et al. [86]
S100A8	Samples from 54 AML patients	predictor of poor prognosis	MS-based proteomics	Nicolas et al. [87]
H3K27me3	Samples from 241 AML patients	predictor of poor prognosis	LC-MS/MS-based proteomics	Djik et al. [97]
FH, IDH2, ENO1, LTF, and GLUL	Serum samples from 51 AML patients	predictor of poor prognosis	TMT-MS/MS-based proteomics	Zhang et al. [98]
ICAM2	BM samples from 10 AML patients and 3 healthy controls	predictor of poor prognosis	LC-MS/MS-based proteomics	Zhang et al. [99]
a 6-metabolite model: lactate, 2-oxoglutarate, pyruvate, 2-HG, glycerol-3-phosphate, and citrate	Serum samples from 134 AML patients for training, 99 for validation	predictor of poor prognosis	MS-based metabolomics	Chen et al. [102]
GLUT5 and SLC2A5	Serum samples from 400 AML patients and 446 healthy controls	predictor of poor prognosis	MS-based metabolomics	Chen et al. [103]
ARA and its precursors	Serum samples from 20 AML patients and 20 healthy controls	predictor of poor prognosis	MS-based metabolomics	Pabst et al. [104]
F2α	Serum samples from 20 AML patients and 20 healthy controls	predictor of favorable prognosis	MS-based metabolomics	Pabst et al. [104]
NUP98::NSD1	Samples from 293 pediatric AML patients and 808 adult AML patients	predictor of chemoresistance and relapse	RNA-seq-based transcriptomics	Hollink et al. [107]
MN1, FHL1, CD34, RBPMS, LPAR6, and NPR3	Blasts from 33 AML patients	predictor of chemoresistance	microarray-based transcriptomics	Heuser et al. [109]
CD28	125 patients from TARGET and 125 from BeatAML	predictor of chemoresistance and relapse	RNA-seq-based transcriptomics	Floren et al. [111]
CD44, HLAs, and PTMA	BM samples from 5 AML patients, 31 AML patients from TARGET, and 16 from Mason	predictor of relapse	scRNA-seq-based transcriptomics	Stetson et al. [118]
a 7-gene model: CLEX11A, PRAME, AZU1, NREP, ARMH1, C1QBP, and TRH	BM samples from 20 AML patients and 301 patients from TARGET for training, 1398 from TARGET for validation	predictor of poor prognosis	scRNA-seq-based transcriptomics	Mumme et al. [120]
Annexin I, γ1 actin	BM samples from 33 AML patients	predictor of chemoresistance	MS-based proteomics	Kaźmierczak et al. [125]
glutathione transferase ω, esterase D	BM samples from 33 AML patients	predictor of complete remission	MS-based proteomics	Kaźmierczak et al. [125]
HMGA1 phosphorylation at CK2 sites	BM samples from 13 AML patients	predictor of chemoresistance	LC-MS/MS-based proteomics and phosphoproteomics	Zhu et al. [126]
BTG1	BM samples from 12 AML patients and 3 healthy controls	biomarker in monitoring the status of complete remission	MS-based proteomics	Cho et al. [130]
phosphorylation of CDKs and CK2		predictor of relapse	LC-MS/MS-based proteomics and phosphoproteomics	Aasebø et al. [131]
V-ATPase proteins	**Cells from 41 AML patients**	predictor of relapse-free	LC-MS/MS-based proteomics and phosphoproteomics	Aasebø et al. [131]
pantothenic acid	Serum samples from 94 AML patients	predictor of chemoresistance	LC-MS-based metabolomics	Stockard et al. [139]
MEF2C	Samples from 47 AML patients	predictor of chemoresistance	RNA-seq and ATAC-seq-based transcriptomics, LC-MS/MS-based phosphoproteomics	Brown et al. [140]
HIF	BM samples from 87 AML patients and 13 healthy controls	predictor of good response to sorafenib	NGS-based transcriptomics	Kivioja et al. [149]
phosphorylation of MAPK, EGFR1, and KIT	BM samples from 35 AML patients	predictor of resistance to FLT3 inhibitors	LC-MS/MS-based proteomics and phosphoproteomics	Cucchi et al. [153]
phosphorylation of S160 in EEPD1, S630 in BCL11A, S333 in RANBP3, S961 in RP3, S458 in LMN1	BM samples from 21 patients for training, 9 for validation	predictor of resistance to FLT3 inhibitors	LC-MS/MS-based proteomics and phosphoproteomics	Schaab et al. [155]
a model based on mRNA and protein levels	Samples from 38 AML patients	predictor of response to FLT3 inhibitor	RNA-seq-based transcriptomics, LC-MS/MS-based phosphoproteomics	Gosline et al. [158]
AURKB	BM samples from 41 AML patients	predictor of early resistance to FLT3 inhibitor	Single-cell targeted DNA-Seq, LC-MS/MS-based proteomics and metabolomics	Joshi et al. [159]
Pim2	Samples of 9 AML patients	predictor of resistance to FLT3 inhibitor	RNA-Seq-based transcriptomics and LC-MS/MS-based proteomics	Hospital et al. [163]
MAC-Score	Samples of 72 AML patients	predictor of response to venetoclax/azacitidine	RNA-Seq-based transcriptomics and LC-MS/MS-based proteomics	Waclawiczek et al. [176]
Mito-AML	Samples from 252 AML patients	predictor of poor prognosis and better response to venetoclax	DNA-Seq-based genomics, RNA-Seq-based transcriptomics, and LC-MS/MS-based proteomics	Jayavelu et al. [177]
DPYSL2	BM samples from 198 AML patients	predictor of poor prognosis	RNA-seq-based transcriptomics	Li et al. [217]
LYPD3	173 AML patients and 337 healthy controls from TCGA	predictor of poor prognosis	RNA-seq-based transcriptomics	Hu et al. [230]
phosphorylation of S315 in p53	Samples from 44 AML patients	predictor of good response to selinexor	LC-MS/MS-based proteomics and phosphoproteomics	Emdal et al. [250]
phosphorylation S253 in FOXO3A	Samples from 44 AML patients	predictor of resistance to selinexor	LC-MS/MS-based proteomics and phosphoproteomics	Emdal et al. [250]

**Table 3:** Novel drug targets in AML identified by omics-based studies

Drug target	Target patients	Sample	Function	Major omics approaches	Literature
BCL6	FLT3-ITD (+)	human and murine AML cell lines, patient-derived AML cells	overcoming gilteritinib resistance	RNA-seq and ATAC-seq-based transcriptomics	Zavorka et al. [88]
DPYSL2	all AML patients	AML cell lines and patient-derived AML cells	finding novel drug target for AML patients	RNA-seq-based transcriptomics	Li et al. [110]
CK2	all AML patients	primary AML blasts from 8 patients and AML cell lines	overcoming resistance to cytarabine	LC-MS/MS-based proteomics and phosphoproteomics	Zhu et al. [126]
Autophagy	FLT3-ITD (+)	AML cell lines and patient-derived primary AML cells	overcoming resistance to the 2nd generation FLT3 inhibitors	LC-MS/MS-based translatome proteomics and phosphoproteomics	Koschade et al. [156]
AURKB	FLT3-ITD (+)	BM samples from 41 primary AML patients and cell lines	overcoming early resistance to FLT3 inhibitors	Single-cell targeted DNA-seq, LC-MS/MS-based proteomics and metabolomics	Joshi et al. [159]
RSK2	FLT3-ITD (+)	9 primary AML patient samples and cell lines	finding potential target for FLT3-ITD (+) AML patients	RNA-seq-based transcriptomics and LC-MS/MS-based proteomics	Hospital et al. [163]
MDM2	all AML patients	AML cell lines and samples from AML mouse model	overcoming resistance to venetoclax	RNA-seq-based transcriptomics	Lehmann et al. [171]
NAMPT	R/R AML patients	AML cell lines and patient-derived AML cells	overcoming resistance to venetoclax/azacitidine	LC-MS/MS-based metabolomics	Jones et al. [175]
Complex I	all AML patients	252 primary AML samples	finding novel drug target for AML patients	DNA-seq-based genomics, RNA-seq-based transcriptomics and LC-MS/MS-based proteomics	Jayavelu et al. [177]
ETFA, ETFB	all AML patients	samples from AML mouse model and TCGA samples	finding novel drug targets for AML and overcoming resistance to venetoclax	RNA-seq-based transcriptomics and LC-MS/MS-based proteomics	Caplan et al. [178]
BET, MOZ, LSD1, and CBP/p300	MLL-r and NPM1 (+)	AML cell lines	overcoming refractory to menin inhibitor	RNA-seq, scRNA-seq, and ATAC-seq-based transcriptomics	Fiskus et al. [184]
ACC1	IDH1 (+)	AML cell lines and patient-derived AML cells	overcoming resistance to ivosidenib	LC-MS/MS-based metabolomics	Bassal et al. [200]
DOT1L	MLL-r	AML cell lines	finding potential drug targets for AML patients with MLL-r	ChIP-seq-based genomics and microarray-based transcriptomics	Guenther et al. [220]
Dock, Rac	NPM1(+) AML	AML cell lines and samples from AML mouse model	finding potential drug targets for AML patients with NPM1 mutation	RNA-seq-based transcriptomics	Meyer et al. [229]
LYPD3	all AML patients	data of 173 AML samples in TCGA	finding novel drug target for AML patients	RNA-seq-based transcriptomics	Hu et al. [230]
ERRα	all AML patients	AML cell lines and patient-derived AML cells	finding novel drug target for AML patients	scRNA-seq-based transcriptomics	Seo et al. [238]
DNA-PK	FLT3-ITD (+)	primary AML blasts from 7 patients and AML cell lines	overcoming midostaurin and sorafenib resistance	LC-MS/MS-based proteomics and phosphoproteomics	Murray et al. [243]
PTK2B	FLT3-ITD (+)	AML cell lines and patient-derived primary AML cells	overcoming early resistance to FLT3 inhibitors	LC-MS/MS-based proteomics	Allert et al. [248]
MDM2, AKT	all AML patients	BM samples from 20 primary AML patients and cell lines	overcoming resistance to Selinexor	LC-MS/MS-based proteomics and phosphoproteomics	Emdal et al. [250]
S100A4	all AML patients	15 primary AML patient samples and AML cell lines	finding novel drug target for AML patients	RNA-seq-based transcriptomics and LC-MS/MS-based proteomics	Alanazi et al. [255]
mitotic kinase	adverse risk AML	74 primary AML patient samples	finding potential drug targets for AML patients with poor prognosis	DNA-seq and NGS-based genomics, RNA-seq-based transcriptomics, and LC-MS/MS-based proteomics and phosphoproteomics	Casado et al. [259]
C1Q	all AML patients	EMI and BM samples from 1 AML patient and samples from AML mouse model	finding drug targets for early-stage treatment of AML	RNA-seq and scRNA-seq-based transcriptomics and LC-MS/MS-based proteomics	Yang et al. [260]
RBM17, EIF4A2	all AML patients	8 primary AML samples, data of 78 AML samples GEO and AML cell lines	finding drugs targeting AML leukemic stem cells	RNA-seq-based transcriptomics and LC-MS/MS-based proteomics	Liu et al. [265].
SIRT3	all AML patients	AML cell lines and patient-derived AML cells	finding drugs targeting AML leukemic stem cells	RNA-seq-based transcriptomics, LC-MS/MS-based proteomics and metabolomics	O’Brien et al. [278].

Among the several omics we discussed above, transcriptomic studies are the most abundant and the most closely related to mutations and cytogenetic abnormalities. Recently, transcriptomic approaches have extensively been used to study numerous fusion genes and various types of expressed patterns in AML. Protein types, expressions, and modifications in AML patients are also well-studied by proteomics approaches. A large number of transcriptomics studies focus on defining subgroups based on GEP and proteomics studies seem to attract interest in drug response prediction and in solving resistance problems. Hence, most multi-omics studies are based on proteo-transcriptomics and mutations, which may well demonstrate the whole process from DNA to protein and provide full insight for AML diagnosis and treatment. As a fresher technique, metabolomics is less conducted and seldom included in multi-omics studies. However, metabolomics has the potential of relaying plenty information about small and minimally invasive samples in a cost-effective way. Metabolomics may play an important role in multi-omics analyses if integrated with other data properly because it provides the immediate readout of response to perturbations like drugs.

### Current challenges and future perspectives

There are some obstacles and challenges in applying omics methods in researches as well as in clinical management of AML. However, we believe that most of them can be solved with rigorous study design and large investment.

### Conceptual changes

The first challenge is the conceptual changes in clinical management of AML. Following a comprehensive and instructive protocol, the management of AML is already systematic. However, precision and personalized medicine is sure to be the future of AML treatment, where accurate molecular diagnosis will be the foundation. Current diagnoses and treatments are still not enough. Conceptual shifts are necessary, especially in frontline hematological physicians, as they are most suitable for exploring ideas for omics studies as well as applying research results to clinical practice. We, therefore, recommend more communication between physicians and researchers to allow more opinions beyond the daily regular treatment of patients. Another challenge in conceptual shift lies in patients. In precision medicine, a comprehensive examination upon diagnosis can be very expensive. One way to settle this is to be patient in explaining the necessity of thorough examinations. An effective way is to cut down the cost in omics studies and omics-related clinical examination. Although the high-cost equipment of omics studies cannot be reduced, up to date, a large set of omics data is now available in public datasets for deeper investigation. Many of the studies we discussed above only demonstrated the RNA or protein expression signature and performed regular statistical analyses to find distinct expression patterns. Further studies can combine information from several different sources of data and perform analyses based on existing conclusions. The cost can be saved and the current omics data can serve as a valuable resource, which can be used by physicians for preliminary analyses.

### Analytical strategies

Analytical challenges of omics data are also unavoidable in omics studies. In AML studies, due to the complexity of the disease, both the disease cases and healthy controls are highly heterogeneous under the influence of population structure, sampling bias, batch effects, bias of cell types, and many other factors. Proper sampling methods and enlargement of sample sizes can help reduce bias, and there are several effective statistical methods to remove batch effects. An unavoidable analytical challenge in addressing omics data is to distinguish causal changes from reactive changes, especially when each dataset is correlative to the other. It is unlikely to distinguish them relying only on one omics data type collected at one-time point. Therefore, we recommend the integration of multi-omics data to solve this problem. Integrated analyses of omics data remain challengeable due to the high dimensionality of the data, including large numbers of identified genes, proteins, modifications, metabolites, and the inter-individual variability among patients. Therefore, we recommend specialized statisticians to undertake the data analysis tasks.

### Translation to clinical use

Finally, it is also difficult to transfer the research results to clinical use. Advances are needed on several fronts before conclusions obtained from these studies can be finally translated to the clinical practice. For diagnostic and prognostic markers, we recommend multi-center studies to eliminate the influence of tumor individual heterogeneity. Moreover, widespread application of the biomarkers in the clinical sphere demands that user-friendly analytical platforms are accessible to personnel from hospitals, and thus convenient measurement procedures are recommended. For novel drug targets, we recommend thorough pre-clinical studies before trials to guarantee patients’ safety in clinical trials. As drug targets need more evidence than biomarkers, large enough sample size and multi-omics information are fundamental to ensure the precision and effectiveness of results. Finally, newly developed drugs are always too expensive. Therefore, we recommend carrying out clinical trials on large scale multi-omics analyses, which can benefit more patients and accelerate the extensive use of these drugs.

In the era of precision medicine, multi-omics-based studies will cover a wide range of areas from diagnosis, and treatment to prognosis, including resistance mechanisms, optimal mode of induction, consolidation, and maintenance therapy, helping identify appropriate therapeutic targets and develop new drugs. Clinical implementation of multi-omics approaches will enable individualized diagnosis and treatment of AML patients by improving classification and therapeutic choices, and will further improve the AML prognoses.

## Data Availability

Data is available from the corresponding author by request.

## Abbreviations

2-HG: 2-hydroxyglutarate
53BP1: Tumor protein p53 binding protein 1
5-FU: Fluorouracil
6-MP: Hydroxyurea, mercaptopurine
A-CGH: Array-based comparative genome hybridization
ACC1: Acetyl-CoA carboxylase 1
ACIN1: Apoptotic chromatin condensation inducer 1
ADAM8: Disintegrin and metalloproteinase domain-containing protein 8
AIFM1: Apoptosis inducing factor mitochondria associated 1
ALL: Acute lymphoblastic leukemia
a-KG: a-ketoglutarate
AMKL: Acute megakaryoblastic leukemia
AML: Acute myeloid leukemia
AML MLD: AML with multilineage dysplasia
APL: Acute promyelocytic leukemia
ARA: Arachidonic acid
Ara-C: Arabinofuranosyl cytidine
ARPC5L: Actin related protein 2/3 complex subunit 5 like
ATAC-Seq: Assay for transposase-accessible chromatin with high-throughput sequencing
ATF-2: Activating transcription factor 2
ATM: Ataxia telangiectasia mutated
AURKB: Aurora kinase B
AZU1: Azurocidin 1
BAD: BCL-2 associated agonist of cell death
BCL-2: B-Cell CLL/lymphoma 2
BET: Bromodomainand extraterminal domain
BFL1: BCL-2 related protein A1
BID: BH3 interacting domain death agonist
BIRC5: Baculoviral IAP repeat containing 5
BM: Bone marrow
BMI: B-cell-specific Moloney murine leukemia virus insertion
BRD4: Bromodomain containing 4
BTG1: B-cell translocation gene 1
C1Q: Complement component 1, Q subcomponent
CASP1: Caspase 1
CBP: CREB-binding protein
CCND1: Cyclin D1
CDK: Cyclin-dependent kinase
CDK6: Cyclin dependent kinase 6
cDNA: Complementary DNA
Cer: Ceramide
CK2: Casein kinase 2
CR: Complete remission
CRADD: CASP2 and RIPK1 domain containing adaptor with death domain
CXCL4: CXC chemokine ligands 4
CyTOF: Mass cytometry by time of flight
DFS: Disease-free-survival
DNA: Deoxyribonucleic acid
DNMT3A: DNA methyltransferase 3a
DNMT3B: DNA methyltransferase 3b
DOCK1: Dedicator of cytokinesis 1
DOT1L: DOT1 like histone lysine methyltransferase
DPYSL2: Dihydropyrimidinase‐like 2
Dx: Diagnosis
E2F: E2 transcription factor
EEPD1: Endonuclease/exonuclease/phosphatase family domain containing 1
EGFR1: Epidermal growth factor receptor-1
EIF4A2: Eukaryotic translation initiation factor 4A2
ELANE: Elastase, neutrophil expressed
ELMO1: Engulfment and cell motility 1
ELN: European LeukmiaNet
EMI: Extramedullary infiltration
EMK: Erythroid megakaryocyte
ENL: Eleven-nineteen leukemia
ENO1: Enolase 1
ERRα: Estrogen-related receptor
ESI-MS/MS: Electron spray ionization-mass spectrometry/mass spectrometry
ETFA: Electron transfer flavoprotein subunit alpha
ETFB: Electron transfer flavoprotein subunit beta
EVI1: Ecotopic viral integration site 1
FAB: Franch, American, Britain
FAM81A: Family with sequence similarity 81 member A
FBXO11: F-box protein 11
FDA: Food and drug administration
FGF2: Fibroblast growth factor 2
FH: Fumarate hydratase
FLT3: Fms related receptor tyrosine kinase 3
FLT3-ITD: Fms-like tyrosine kinase 3-internal tandem duplication
FLT3-TKD: Fms-like tyrosine kinase 3-tyrosine kinase domain
FOXO3A: Forkhead box O3
GATA2: GATA binding protein 2
GEP: Gene expression profiling
GLUL: Glutamate-ammonia ligase
GLUT5: Solute carrier family 2 member 5
GSEA: Gene set enrichment analysis
GZMB: Granzyme B
HDL: High-density lipoprotein
HMGA1: High mobility group AT-hook 1
HMGB1: High mobility group box 1
HMG-CoA: Hydroxy methylglutaryl coenzyme A
HMGN2: High mobility group nucleosomal binding protein 2
hnRNPH1: Heterogeneous nuclear ribonucleoprotein H1
HOXA: Homeobox A
HOXB: Homeobox B
HSC: Hematopoietic stem cell
IC_50_: Inhibitory concentration
ICAM2: Intercellular adhesion molecule-2
IDH1: Isocitrate dehydrogenase (NADP(+)) 1
IDH2: Isocitrate dehydrogenase (NADP(+)) 2
IFN-γ: Interferon-γ
IL12: Interleukin-12
IMP: Immature progenitor
IMPDH: Inosine monophosphate dehydrogenase
INKA: The integrative inferred kinase activity
IPSS: International prognostic scoring system
IPSS-R: The revised international prognostic scoring system
IRF: Interferon regulatory factor
ITGAM: Integrin subunit aM
JAK2: Janus kinase 2
JMJD1C: Jumonji domain containing 1C
KIT: Receptor tyrosine kinase Kit
KLF6: Kruppel like factor 6
KMT2A: Lysine methyltransferase 2A
KPNA4: Karyopherin subunit a4
KPNB1: Karyopherin subunit b1
KSEA: Kinase substrate enrichment analysis
LAMP5: Lysosomal associated membrane protein family member 5
LAPTM5: Lysosomal-associated multispanning membrane protein-5
LASSO: Least absolute shrinkage and selection operator
LC–MS: Liquid chromatography–mass spectrometry
LDL: Low-density lipoprotein
LICs: Leukemia-initiating cells
LIMK: LIM domain kinase
LMN1: Lamin A/C
LOXL1: Lysyl oxidase like 1
LPXN: Leupaxin
LSCs: Leukemia stem cells
LSD1: Lysine specific demethylase1
LTF: Lactotransferrin
LYPD3: Ly6/PLAUR domain-containing protein 3
LYZ: Lysozyme
MAC-Score: Mediators of apoptosis combinatorial score
MADS: MCM1agamous-deficiens-serum response factor
MALDI-TOF-MS: Matrix-assisted laser desorption/ionization time of flight mass spectrometry
MAPK: Mitogen-activated protein kinase
MCL1: Myeloid cell leukemia 1
MDMX: Murine double minute X
MDS: Myelodysplastic syndrome
MDS-AML: MDS-related AML
MEF2C: Myocyte enhancer factor 2C
MEK/ERK: Mitogen-activated protein kinase kinase/extracellular signal-regulated kinase
Mito-AML: Mitochondrial protein expression
MLL: Mixed lineage leukemia
MOFA: Multi-omics factor analysis
MOZ: Lysine acetyltransferase 6A
MR: AML myelodysplasia-related
MRD: Minimal residual disease
MS: Mass spectrometry
mTOG: Terminal oligoguanine
mtOXPHOS: Mitochondrial oxidative phosphorylation
NAP1L1: Nucleosome assembly protein 1-like 1
NK: Normal karyotype
NK AML: AML samples with normal karyotype
NPM1: Nucleophosmin 1
OS: Overall survival
P13K: Phosphoinositide 3-kinase
PABPC1: Polyadenylate-binding protein cytoplasmic 1
PAIP1: PABPC1-interacting proteins 1
PBX3: PBX homeobox 3
PCA: Principal component analysis
PCSF: Prize collecting steiner forest
PEAR1: Amyloid b precursor like protein 1
PF4: Platelet factor 4
PGF2a: Prostaglandin F2a
POU4F1: POU class 4 homeobox 1
PRTN3: Proteinase 3
PTK2B: Protein tyrosine kinase 2b
PTMA: Prothymosin a
PTMs: Post-translational modifications
R/R: Relapse/refractory
RAEB: Refractory anemia with excess of blasts
RANBP3: RAN binding protein 3
RARG: Retinoic acid receptor g
RAS: Rat sarcoma viral oncogene homolog
RCMD: Refractory cytopenia with multilineage dysplasia
Re: Relapse
RFS: Relapse-free survival
RMB-17: RNA-binding motif protein 17
RNA-seq: Ribonucleic acid sequencing
ROS: Reactive oxygen species
RP3: Retinitis pigmentosa GTPase regulator
RPPA: Reverse-phase protein array
RPS6KA3: Ribosomal protein S6 kinase A3
RRM: RNA-recognition motif
RSK2: Ribosomal protein S6 kinase A3
RTKs: Receptor tyrosine kinases
RUNXI: RUNX family transcription factor 1
sAML: Secondary acute myeloid leukemia
scRNA-seq: Single-cell ribonucleic acid sequencing
SELDI: Surface-enhanced laser desorption ionization
SELDI-TOF MS: Surface-enhanced laser desorption/ionization time-of-flight mass spectrometry
SENP6: SUMO specific peptidase 6
SF3B1: Splicing factor 3B subunit 1
SILAC: Stable isotope labeling with amino acids in cell culture
SIRT1: Sirtuin 1
SLC2A5: Solute carrier family 2 member 5
SM: Sphingomyelin
SMC1A: Structural maintenance of chromosomes 1A
SNP-As: Single-nucleotide polymorphism arrays
SPTAN1: Spectrin a, non-erythrocytic 1
SRSF2: Serine/arginine-rich splicing factor 2
STAG2: Stromal antigen 2
STAT5A: Signal transducer and activator of transcription 5A
TALDO1: Transaldolase 1
TCGA: The cancer genome atlas research network
TKIs: Tyrosine kinase inhibitors
TNFα: Tumor necrosis factor α
TPI1: Triosephosphate isomerase 1
TPOR: MPL proto-oncogene
tRFs: Transfer RNA -derived fragments
TRL: Therapy-related leukemia
U2AF1: U2 snRNP auxiliary factor small subunit
UBE3B: Ubiquitin-protein ligase E3B
ULK1: Unc-51 like autophagy activating kinase 1
USP9X: Ubiquitin specific protease 9, X chromosome
VEGF: Vascular endothelial growth factor
VLDL: Very low density lipoprotein
WBC: White blood cell
WGCNA: Weighted gene co-expression network analysis
WHO: World Health Organization
XPO1: Nuclear export protein exportin-1
XRCC: X-ray repair cross complementing
